# Computed tomography-based prediction of early recurrence risks with estimating individual times to recurrence for lung cancer patients prior to radiotherapy

**DOI:** 10.1016/j.phro.2026.101021

**Published:** 2026-06-13

**Authors:** Takumi Kodama, Hidetaka Arimura, Yuko Shirakawa, Yagiz Yedekci, Hidetake Yabuuchi, Yu Jin, Noriyuki Nagami, Tadamasa Yoshitake, Yoshiyuki Shioyama, Pervin Hurmuz

**Affiliations:** a Division of Medical Quantum Science, Department of Health Sciences, Graduate School of Medical Sciences, Kyushu University, 3-1-1 Maidashi, Higashi-ku, Fukuoka 812-8582, Japan; b Division of Medical Quantum Science, Department of Health Sciences, Faculty of Medical Sciences, Kyushu University, 3-1-1 Maidashi, Higashi-ku, Fukuoka 812-8582, Japan; c Joint Graduate School of Mathematics for Innovation, Kyushu University, 744 Motooka, Nishi-ku, Fukuoka, 819-0395, Japan; d Radiology Informatics and Network, Graduate School of Medical Sciences, Kyushu University, 3-1-1 Maidashi, Higashi-ku, Fukuoka 812-8582, Japan; e Department of Radiation Oncology, Faculty of Medicine, Hacettepe University, Hacettepe Mahallesi, 06230 Sıhhiye, Ankara, Turkey; f Department of Radiology, Saga University Hospital, 5-1-1, Nabeshima, Saga City, Saga 849-8501, Japan; g Ion Beam Therapy Center, SAGA-HIMAT Foundation, 3049 Harakoga-machi, Tosu, Saga 841-0071, Japan

**Keywords:** Time to recurrence, Bayesian approach, Radiomics, Non-small cell lung cancer, Radiotherapy

## Abstract

**Background and Purpose:**

Current radiomics approaches have limitations in predicting recurrence with the time gap between radiomic features prior to treatment and individual recurrence risks following treatment. This study aimed to develop computed tomography (CT)-based prediction models of early recurrence risks by estimating individual times to recurrence (TTRs) in patients with non-small cell lung cancer (NSCLC) prior to stereotactic ablative radiotherapy (SABR).

**Materials and methods:**

A total of 143 patients with stage I-II NSCLC treated with SABR were enrolled. Planning CT images were used to construct internal (*n* = 125; 43 recurrent, 56 censored, and 26 recurrence-free within 5 years) and external (*n* = 18; 15 recurrent and 3 recurrence-free) datasets. Reference TTR data from 124 recurrent patients were used to estimate virtual TTRs of 56 censored patients using a Bayesian approach with the Markov chain Monte Carlo algorithm. Three types of regression models were developed to estimate the TTR based on significant CT image features associated with early recurrences. The predictions of 1-year and 2-year recurrences based on the estimated TTRs were evaluated using the areas under the receiver operating characteristic curves (AUCs).

**Results:**

The best prediction models achieved AUCs of 0.926 and 0.763 for 1-year and 0.869 and 0.883 for 2-year recurrence in the internal and external tests, respectively.

**Conclusion:**

The proposed CT-based model demonstrated improved performance for predicting 1- and 2-year recurrences by directly predicting TTRs. This study could bridge the time gap between pretreatment radiomic features and individual recurrence risks.

## Highlights

• Bayesian approach estimates virtual time to recurrence for censored patients.
• Proposed model predicted early recurrences by directly predicting time to recurrence.
• This study could bridge the time gap between pretreatment images and recurrence risks.

## Graphical abstract

**Figure:**
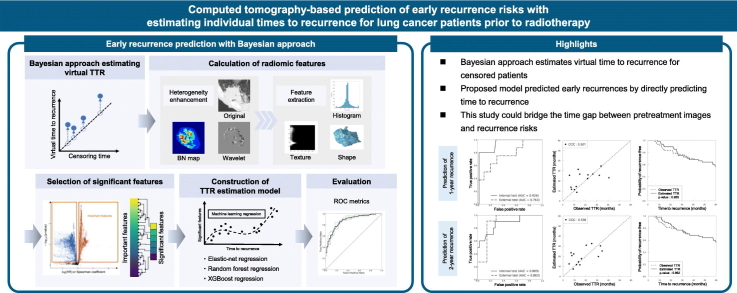

## 1 Introduction

Stereotactic ablative radiotherapy (SABR) is a standard treatment option for medically inoperable patients or those unwilling to undergo surgery with stage I-II non-small cell lung cancer (NSCLC) [1], with overall survival comparable to surgical resection [2], [3], [4]. However, the rates of locoregional recurrence and distant metastasis are 12.8 and 22.1%, respectively, within 3 years after SABR [4], and the corresponding 5-year rates are 25.5 and 23.6%, respectively [5]. Senthi et al. [6] reported that the median TTR, defined as time from treatment to any local, regional, or distant recurrence, was 11.4 months. Therefore, the prediction of early individual recurrence risk is highly important to further reduce the number of recurrent patients after SABR.

Previous studies [7], [8] investigated computed tomography (CT)-based recurrence prediction in NSCLC patients prior to SABR without considering TTRs. The studies [7], [8] investigated stratification of patients into high-risk and low-risk groups for locoregional recurrence and distant metastasis using radiomics machine learning models. However, there were time gaps in predicting recurrence between radiomic (CT image) features prior to treatment and individual recurrence risks following treatment.

To bridge the gaps, the estimation of TTRs could be helpful for predicting recurrence. To our knowledge, no studies have reported the direct estimation of TTRs in NSCLC patients prior to SABR. This study aimed to develop CT-based prediction models of early recurrence risks through estimation of patients' TTR.

However, two major challenges remained: the number of recurrent patients was small, and there were censored patients whose TTRs were unknown. For censored patients, the TTRs are considered to exceed the censoring time [9], and censored patients may have higher prognostic risks of recurrence [10], [11], [12], [13]. Therefore, approaches to estimate TTRs for censored patients were needed to augment the training cohort with virtual TTRs.

Moghaddam et al. [14] developed a Bayesian imputation approach for censored survival data using their own internal dataset as reference. However, independent reference data would be preferable to increase the generalization of estimated TTRs. Therefore, we developed a novel Bayesian approach for estimating virtual TTRs of censored patients using independent reference TTRs [6], which may facilitate the development of TTR prediction models.

We hypothesized that individual TTR estimates for NSCLC patients could bridge the gap in radiomics between CT image features prior to treatment and individual recurrence risks following treatment. We proposed CT-based prediction models of early (1 and 2 years) recurrence risks by estimating individual TTRs in NSCLC patients prior to SABR. Furthermore, we investigated training data augmentation using the Bayesian approach to estimate virtual recurrences for censored patients based on independent reference data.

## 2 Materials and methods

### 2.1 Study design

This study used clinical information from patients with stage I–II NSCLC treated with SABR at Kyushu University Hospital (KUH; *n* = 125) and Hacettepe University Hospital (HUH; *n* = 18). Patients from the VU University Medical Center (VUC; *n* = 124) were employed as a reference TTR distribution [6]. This study was approved by the institutional review boards of KUH (22307–00) and HUH (16969557) and conducted in accordance with the Declaration of Helsinki.

**Fig. 1:**
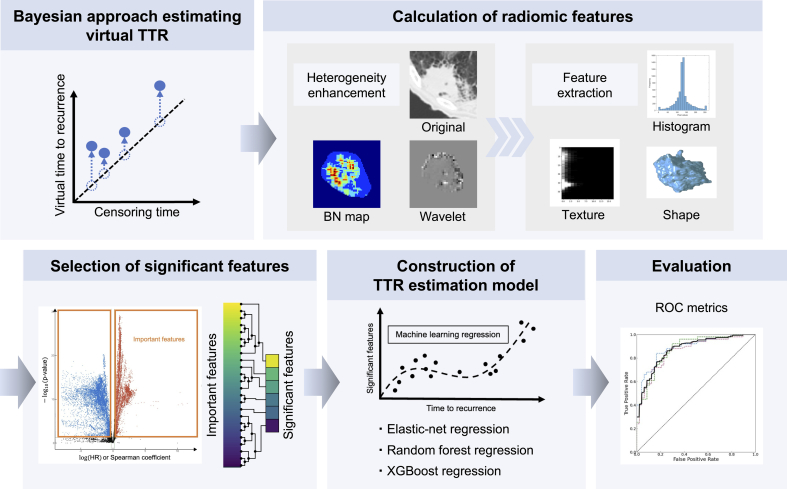
Framework for predicting early recurrence risks by estimating time to recurrence (TTR). BN map: Betti number map, HR: Cox hazard ratio, XGBoost: extreme gradient boosting, ROC: receiver operating characteristic.

Fig. 1 shows the framework for predicting early recurrence risks by estimating the individual TTR. The detailed workflow and execution environment are described in Supplementary Fig. S1 and Document S1, respectively.

### 2.2 Definitions of recurrent, censored, and recurrence-free patients

TTR was defined as the time to any local, regional, or distant recurrence within 5 years from treatment [6]. Any other events censoring the 5-year follow-up, such as death from non-cancer causes or loss to follow-up, were treated as right-censoring. Based on these definitions, patients were classified into recurrent (who experienced any recurrences within 5 years), censored (whose follow-up was right-censored within 5 years), and recurrence-free (who remained free of any recurrences within 5 years) patients.

### 2.3 Clinical information

This study included 267 patients with stage I–II NSCLC treated with SABR. A total of 125 patients (43 recurrent, 56 censored, and 26 recurrence-free) from KUH (treated between May 2004 and October 2016) were used as the internal dataset, and 18 patients (15 recurrent and 3 recurrence-free) from HUH (treated between Aug 2018 and Aug 2021) were used as the external dataset. The external test dataset was intentionally enriched with recurrent patients to ensure a sufficiently broad range of TTRs in an independent setting. The reasons for censoring in the KUH dataset were death from other diseases in 12 patients and lack of follow-up information in 44 patients due to transfer to another hospital or unexpected dropout. Table 1 shows the clinical information of the KUH and HUH datasets. The KUH patients were re-staged according to the UICC 8th edition, under which pure ground-glass nodules smaller than 3 cm were reclassified as Stage 0 (Tis) (*n* = 10). All Stage II patients had N0 or Nx. A reference TTR distribution of 124 patients from the VUC (treated between April 2003 and December 2011) [6] was used in the Bayesian approach. Supplementary Table S1 summarizes the dose prescription of the three datasets.

**Table 1:** Clinical information of patients in the internal dataset from Kyushu University Hospital (KUH) and the external dataset from Hacettepe University Hospital (HUH). n: number of patients.

	KUH (n = 125)	HUH (*n* = 18)
Age [year, min - max (median)]	60–91 (78)	64–83 (73)
Sex		
Male	90	14
Female	35	4
Stage (UICC 8th edition)		
0	10	0
IA1 / IA2 / IA3 / IB	3 / 34 / 41 / 22	3 / 5 / 5 / 3
IIA / IIB	14 / 1	1 / 1
Histopathology		
Adenocarcinoma	74	5
Squamous cell carcinoma	42	9
Large cell carcinoma	4	0
Unknown	5	4
Prognosis		
Recurrence within 5 years	43	15
Local recurrence	26	8
Regional recurrence	15	8
Distant metastasis	21	12
Censored observation	56	0
Recurrence-free within 5 years	26	3

### 2.4 Bayesian approach for estimating virtual time to recurrence for censored patients

**Fig. 2:**
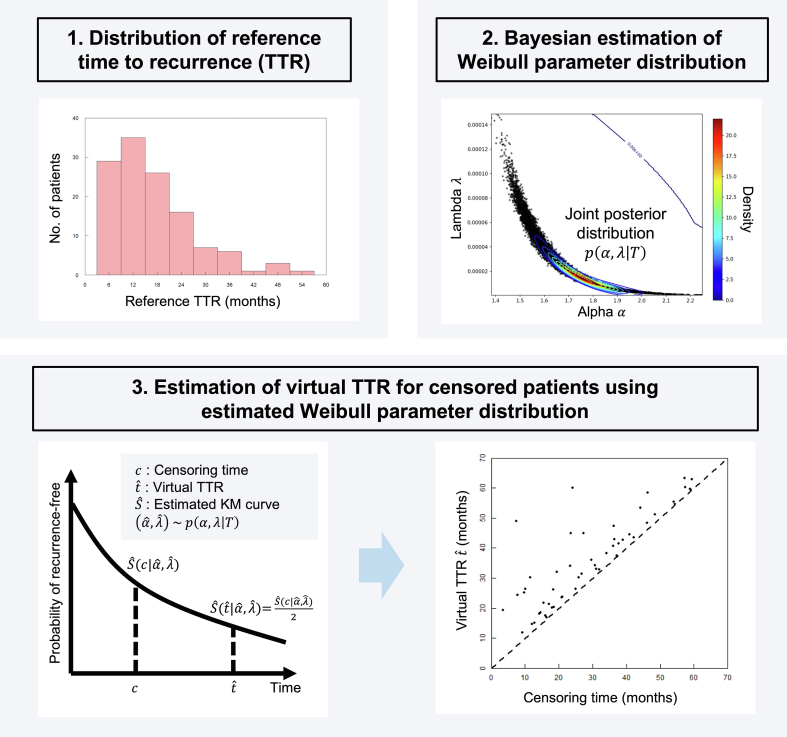
Algorithm of the Bayesian approach for estimating virtual time to recurrence (TTR) based on reference TTR. KM: Kaplan-Meier.

Virtual TTRs for the censored patients were estimated from censoring times and Weibull parameter distributions derived using a Bayesian approach (Fig. 2 and Document S2). The Weibull parameter distributions were derived as a joint posterior distribution given reference TTRs using Bayesian estimation with a Markov chain Monte Carlo (MCMC) algorithm.

The reference TTR distribution was obtained from a TTR histogram [6] of NSCLC patients treated with SABR at the VUC and assumed to follow a Weibull distribution [15]. The Bayesian estimation was performed using the Gibbs sampling method, an MCMC algorithm. The conditional density distribution f and conditional probability S of recurrence-free for TTR t are represented as follows:

(1) $$f\left(t|\alpha ,\lambda \right)=\alpha \lambda t^{\alpha -1}\exp\left(-\lambda t^{\alpha }\right)$$

and

(2) $$S\left(t|\alpha ,\lambda \right)=\exp\left(-\lambda t^{\alpha }\right)$$

where t is the TTR, α and λ are the shape and shift parameters, respectively, of the Weibull distribution. The joint posterior distribution $p\left(\alpha ,\lambda |T\right)$ given a reference TTR T is based on Bayes' theorem as follows:

(3) $$p\left(\alpha ,\lambda |T\right)\propto L\left(T|\alpha ,\lambda \right)\pi \left(\alpha \right)\pi \left(\lambda \right)$$

where L is a likelihood function derived from the conditional density function f (Eqs. (S2–7) and (S2–8) in Document S2), and $\pi \left(\alpha \right)$ and $\pi \left(\lambda \right)$ are prior probability functions (Eqs. (S2–4) and (S2–5) in Document S2). The Gibbs sampling provides the joint posterior distribution $p\left(\alpha ,\lambda |T\right)$ (Figs. 2 and S2).

The virtual TTR *t^* was estimated from the median of the conditional recurrence-free probability $S\left(t|t\geq c,\alpha ,\lambda \right)=0.5$ [14], considering the censoring time c, as shown in Eq. (4).

(4) t^i=ln2λ^i+ciα^iα^i

where

(5) $$\left(\hat{\alpha_{i}},\hat{\lambda_{i}}\right)∼p\left(\alpha ,\lambda |T\right)$$

i represents the censored patient number and ∼ indicates the sampling. The parameters $\left({\hat{\alpha }}_{i},{\hat{\lambda }}_{i}\right)$ for each censored patient i were sampled from the joint posterior distribution $p\left(\alpha ,\lambda |T\right)$ of Eq. (5). Censored patients (*n* = 56) were classified into virtually recurrent (virtual TTR < 5 years; *n* = 49) and recurrence-free (virtual TTR ≥ 5 years; *n* = 7) patients.

### 2.5 Data splitting

The KUH internal patients were divided into three groups: real recurrent (*n* = 43), virtual recurrent (n = 49), and recurrence-free (*n* = 33) patients. The real recurrent patients were divided into three equivalent cohorts (Table S2) to perform a three-fold cross-validation test according to the transparent reporting of a multivariable prediction model for individual prognosis or diagnosis (TRIPOD) recommendations [16]. The training dataset was constructed with the real recurrent cohort and virtual recurrent patients, the validation dataset was the real recurrent cohort, and the internal test dataset consisted of the real recurrent cohort and recurrence-free patients (Fig. S3). The numbers of event and event-free patients in 1-year and 2-year recurrence predictions are summarized in Table S3. The data splitting without the Bayesian approach is shown in Fig. S4.

### 2.6 Image information and preprocessing

Pretreatment planning CT images and gross tumor volumes (GTVs) used for treatment planning were used to extract radiomic features. Table S4 shows the CT image information for the KUH and HUH patients. The anisotropic CT images and GTVs were preprocessed by isovoxelization, Laplacian of Gaussian filtering, and re-quantization to q bits before extraction of radiomic features (Document S1).

### 2.7 Extraction of radiomic features

Three types of radiomic features were calculated from the preprocessed CT (original) images, wavelet-decomposed images, and Betti number (BN) maps. Original features were calculated from the original images using 18 histogram, 75 texture, and 14 three-dimensional (3D) shape-based feature extraction methods (Table S5). The wavelet features were calculated from 3D wavelet-decomposed CT images [7] using histogram and texture methods. The BN map features were calculated from six types of BN maps [[7], [17]] using the histogram and texture methods. All image features were normalized to z-scores determined by the means and standard deviations of the training dataset.

### 2.8 Synthetic minority over-sampling technique augmentation

The virtual TTR estimated using censoring time was longer than the observed TTR (Eq. (4)). Therefore, a synthetic minority over-sampling technique (SMOTE) simultaneously augmented real recurrent patients with TTRs and radiomic features, and an augmented training dataset (*n* = 98) was obtained to balance the numbers of real and virtual recurrent patients. It was confirmed that negative TTR values were not generated by the augmentation.

### 2.9 Selection of significant features

Six significant features (approximately 10% of the number of training patients) were selected using Cox hazard ratio (HR) and Spearman correlation coefficient (SC) based selection methods from important features using hierarchical clustering and ranking of importance distances on volcano plots [[18], [19], [20]]. The workflow is illustrated in Fig. S5. Important features were determined by thresholding adjusted *p*-values and either the HR or SC on the volcano plot. The p-values were adjusted using the Benjamini-Hochberg procedure, and importance distances were calculated using the Euclidean distance from threshold coordinates (Document S3). The significant features had the longest importance distance in each cluster determined by hierarchical clustering using Ward's minimum variance method. The detailed algorithm is described in Document S3.

### 2.10 Construction of TTR estimation models

Three types of machine learning regression models to estimate the natural logarithm of the TTR were constructed using elastic-net, random forest, and extreme gradient boosting. The TTRs were estimated on a logarithmic scale to avoid negative values, and the outputs were exponentially converted to the original scale before comparison with the real TTRs. The hyperparameters (Table S6) were optimized by maximizing the robustness index (RI) in 100 iterations of Bayesian optimization [21]. The RI was calculated from the concordance correlation coefficients (CCCs) [22] in the training ${\mathrm{CCC}}_{\mathrm{train}}$ and validation ${\mathrm{CCC}}_{\mathrm{valid}}$ as follows:

(6) $$\mathrm{RI}=\frac{\mathrm{CC}C_{\text{valid}}}{1+\left|\mathrm{CC}C_{\text{train}}-\mathrm{CC}C_{\text{valid}}\right|}$$

The most robust combination of radiomic features, selection model, and regression model was determined by the mean CCC across all three folds of the internal test dataset (Table S11). The distributions of observed and estimated TTRs were compared using Kaplan-Meier (KM) curves.

### 2.11 Evaluation of the proposed prediction models

Early recurrences within 1 year (1-year recurrence) and 2 years (2-year recurrence) were predicted by thresholding the estimated TTRs and evaluated using areas under the receiver operating characteristic (ROC) curves (AUCs), accuracy, sensitivity, and specificity. Patients with estimated TTRs less than 1 and 2 years were classified as 1- and 2-year recurrent, respectively. The prediction models with and without the Bayesian approach were compared. Cox regression and random survival forest models were also implemented as conventional methods (Document S4). The ROC metrics of the best combination for each radiomic feature type in the internal test dataset are summarized in Table 2. The best representative models were selected based on the highest internal test AUC and evaluated on the independent external test dataset. A sensitivity analysis was performed by varying the number of virtual recurrent patients in the training from 0 to 49 in steps of 5, with 1000 randomly sampled combinations for each number. Furthermore, using the uniform manifold approximation and projection (UMAP) method [23], the association between significant feature vectors and TTRs was investigated.

**Table 2:** Mean receiver operating characteristic (ROC) metrics of 1-year and 2-year recurrence predictions in the training, validation, and internal test datasets of the three-fold cross-validation test. BN map: Betti number map, HR: Cox hazard ratio, SC: Spearman correlation coefficient, EN: elastic net, RF: random forest, CR: Cox regression, RSF: random survival forest, AUC: area under the ROC curve, ACC: accuracy, SEN: sensitivity, SPE: specificity.

				Training	Validation	Internal test			
Early recurrence	Features	Bayesian approach	Selection – estimation models	AUC	AUC	AUC	ACC	SEN	SPE
1-year recurrence	Original	w/	HR - RF	0.943	0.808	**0.851**	0.908	0.356	0.984
	Wavelet	w/	HR - RF	0.956	0.820	0.839	**0.915**	0.356	**0.992**
	BN map	w/	HR - RF	0.937	0.789	0.808	0.908	0.356	0.980
	Original	w/o	SC - EN	0.888	0.785	0.812	0.866	0.478	0.919
	Wavelet	w/o	HR - EN	0.906	0.810	0.799	**0.915**	0.600	0.960
	BN map	w/o	HR - RF	**0.981**	0.798	0.780	0.803	0.644	0.823
	Original	w/o	HR – CR	0.788	0.836	0.744	0.467	**1.00**	0.111
	Original	w/o	HR - RSF	0.858	**0.847**	0.758	0.562	**1.00**	0.269
2-year recurrence	Original	w/	HR - RF	0.873	0.723	**0.802**	0.698	0.700	0.697
	Wavelet	w/	HR - RF	0.900	0.799	0.745	**0.767**	0.533	**0.830**
	BN map	w/	HR - RF	0.912	**0.901**	0.753	0.726	0.600	0.760
	Original	w/o	SC - EN	0.872	0.684	0.734	0.367	0.833	0.243
	Wavelet	w/o	HR - EN	0.821	0.676	0.739	0.260	**1.00**	0.060
	BN map	w/o	HR - RF	**0.931**	0.813	0.684	0.438	0.767	0.350
	Original	w/o	SC – CR	0.772	0.839	0.674	0.675	0.967	0.00
	Original	w/o	HR – RSF	0.801	0.808	0.794	0.698	**1.00**	0.00

## 3 Results

Baseline characteristics of recurrent, censored, and recurrence-free patients in the internal KUH dataset are summarized in Table S7. Fig. S6 shows the distributions of estimated virtual TTR and censoring time of the censored patients and real TTR of recurrent patients.

Table 2 shows the mean AUC, accuracy, sensitivity, and specificity for 1-year and 2-year recurrence predictions in the three-fold cross-validation test. The highest mean AUCs in the internal test were 0.851 and 0.801 for 1-year and 2-year recurrence predictions of the original feature-based models with the Bayesian approach. The ROC metrics for the individual models are presented in Tables S8 and S9.

**Fig. 3:**
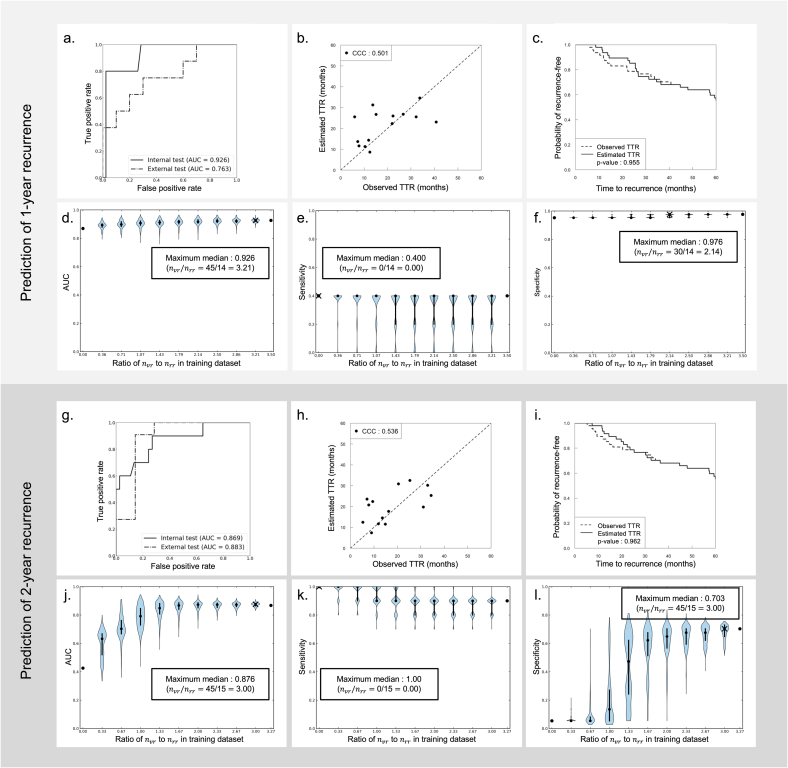
Predictability of the best original feature-based models with the Bayesian approach for 1-year and 2-year recurrence predictions of individual time to recurrence (TTR) estimation. (a, g): Receiver operating characteristic (ROC) curves of the 1-year and 2-year recurrence predictions in the internal and external tests. (b, h): Scatter plots illustrating the individual observed and estimated TTRs for real recurrent patients in the internal test. (c, i): Kaplan-Meier curves comparing the distributions of observed and estimated TTRs in the internal test. (d-f, j-l): Violin plots representing sensitivity analysis in the internal test. The cross mark indicates the maximum median metric. CCC: concordance correlation coefficient, AUC: area under the ROC curve, $n_{\mathrm{vr}}$: number of virtual recurrent patients, $n_{\mathrm{rr}}$: number of real recurrent patients.

The best models achieved AUCs of 0.926 and 0.763 for 1-year and 0.869 and 0.883 for 2-year recurrence in the internal and external tests, respectively (Table S10). Figs. 3(a) and 3(g) show the ROC curves of the best prediction models for the internal and external tests.

Figs. 3(b) and 3(h) are scatter plots illustrating the observed and estimated TTRs for real recurrent patients in the internal test. The models showed CCCs of 0.501 and 0.536 for 1-year and 2-year recurrences, respectively (Table S11).

Figs. 3(c) and 3(i) show KM curves comparing the observed and estimated TTR distributions. There were no statistically significant differences (*p* > 0.05) in the KM curves of all radiomic feature-based models (Table S12).

Figs. 3(d-f), 3(j-l), and S7 show the sensitivity analysis of the best prediction models. As the number of virtual recurrent patients increased, the balance between sensitivity and specificity improved.

**Fig. 4:**
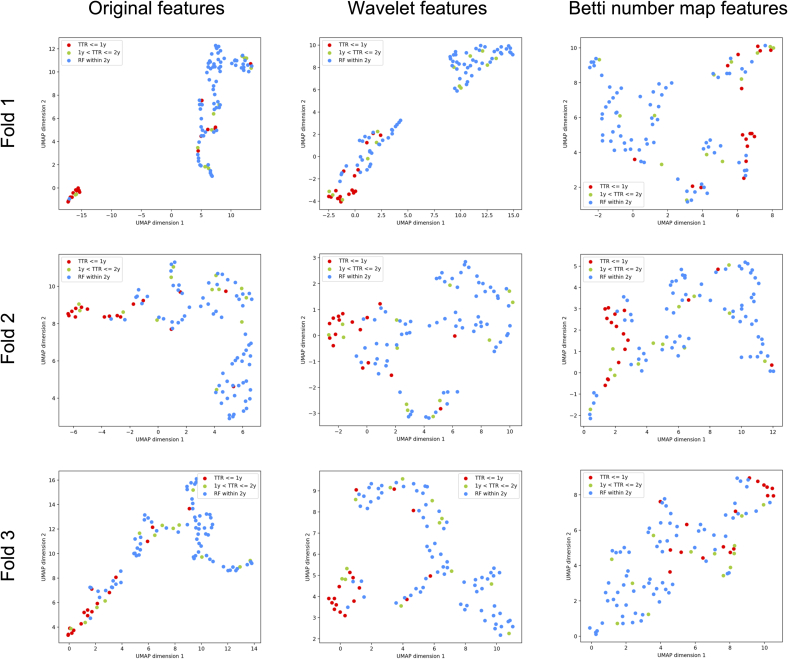
Uniform manifold approximation and projection (UMAP) visualization of the distribution of significant features based on three time to recurrence (TTR) conditions: early (TTR ≤ 1 year), middle (1 year < TTR ≤ 2 years), and longer (recurrence-free within 2 years) TTR groups.

Fig. 4 shows the UMAP of significant features (Table S13) based on TTR conditions. The correlations between significant features and TTR are shown in Fig. S8.

## 4 Discussion

This study proposed a Bayesian approach to estimate virtual recurrence for censored patients based on independent reference data. The TTR estimation model trained with virtual recurrent patients showed higher predictive performance than that trained without them (Table 2). The Bayesian approach may be useful for imputing right-censored survival data for machine-learning models.

In this study, the Weibull distribution was assumed for the reference TTR distribution based on previous reports demonstrating its adequate fit to survival data in NSCLC and other cancer types [15], [24]. Although alternative parametric distributions such as Lognormal and Gamma distributions may also provide adequate fits, the comparison of parametric distributions for modeling TTR remains an area for future investigation.

The proposed Bayesian approach incorporates the information that censored patients were recurrence-free until their censoring time, consistent with Kaplan-Meier analysis. As shown in Fig. S6, the virtual TTR distribution is shifted toward longer times (Eq. 4). However, the current approach does not reflect censoring reasons or clinical characteristics, and further refinement is required.

Table S7 compares the baseline characteristics of the three patient groups. Significant differences in age, sex, and stage suggest associations with censoring risk, recurrence risk, and recurrence-free status, respectively. Integration of clinical information with radiomic features remains an important direction for future studies.

The sensitivity analysis investigated the effect of the number of virtual recurrent patients on model performance. The AUC in the internal test showed a gradual increase followed by a plateau at approximately a ratio greater than 2.0 of the number of virtual recurrent patients to that of the number of real recurrent patients (Figs. 3 and S7). As the ratio increased toward the plateau range, the sensitivity and specificity became more balanced. When virtual recurrent patients were not included, the model showed a relatively high AUC but with an imbalance between sensitivity and specificity, suggesting overfitting to the limited number of real recurrent patients. These findings support the validity of the Bayesian imputation approach as a data augmentation strategy for TTR estimation model training.

The estimated TTR-based prediction of early recurrences showed higher AUCs in the internal and external tests (Fig. 3 and Table S10). Luo et al. [25] and Gao et al. [26] predicted local recurrence and distant metastasis within 1 year with AUCs of 0.818 and 0.71–0.70, respectively. The proposed method achieved AUCs of 0.926 and 0.763 in the internal and external tests, respectively, for 1-year recurrence prediction, and also provided individual estimated TTRs representing a time-dependent recurrence risk. Future studies should investigate the prediction of times to locoregional recurrence and distant metastasis independently.

In Fig. 4, the UMAP demonstrates clustering according to temporal recurrence status based on TTRs in the significant original features. In particular, patients with 1-year recurrence formed clearly separated clusters. The significant original features showed significant Spearman correlations with the observed TTR for real recurrent patients (Fig. S8). Therefore, significant radiomic features could capture tumor phenotypic signals associated with SABR outcomes and may serve as imaging biomarkers for future clinical trials.

This study had five limitations. First, the datasets were intentionally designed with class imbalance: the training dataset consisted predominantly of recurrent patients, while the external test dataset was enriched with recurrent patients. This limits the reliability of specificity and calibration assessment, and the primary purpose of these tests was to evaluate the model's discriminability with a sufficient variety of TTRs. External validation using a natural larger cohort will be necessary. Second, the proposed Bayesian approach did not consider competing risks for the censoring reasons, which may have introduced bias into the estimated virtual TTRs. More sophisticated approaches accounting for differing censoring mechanisms are required. Third, the SABR treatment protocols were not identical across the three institutions (Table S1), and the higher biologically effective dose in a proportion of reference data may have influenced the virtual recurrence estimation. Future studies should use a reference TTR distribution from a cohort with more similar treatment protocols. Fourth, the internal test dataset was used for both model selection and evaluation, which may introduce a slight optimistic bias for the internal test metrics. In contrast, the external test dataset and metrics were completely independent of the model selection process. Fifth, the C-index and Kaplan-Meier curves with *p*-values (Table S12) were evaluated with the reference event status shared between the observed and estimated TTRs, because the proposed model does not identify patients' recurrence status. Therefore, these metrics should be interpreted as reference metrics for comparing TTR estimation accuracy.

In conclusion, the proposed CT-based model demonstrated improved performance in predicting 1- and 2-year recurrences by directly predicting TTRs. The proposed models could bridge the gap between pretreatment radiomic features and individual recurrence risks by estimating individual TTRs.

## CRediT authorship contribution statement

**Takumi Kodama:** Writing – review & editing, Writing – original draft, Visualization, Validation, Software, Resources, Project administration, Methodology, Investigation, Funding acquisition, Formal analysis, Data curation, Conceptualization. **Hidetaka Arimura:** Writing – review & editing, Writing – original draft, Visualization, Validation, Supervision, Resources, Project administration, Methodology, Investigation, Funding acquisition, Data curation, Conceptualization. **Yuko Shirakawa:** Resources, Data curation, Conceptualization. **Fazli Y. Yedekci:** Validation, Resources, Data curation. **Hidetake Yabuuchi:** Validation, Data curation. **Yu Jin:** Validation, Methodology. **Noriyuki Nagami:** Validation, Methodology. **Tadamasa Yoshitake:** Supervision, Resources, Data curation. **Yoshiyuki Shioyama:** Resources, Data curation. **Pervin Hurmuz:** Writing – review & editing, Resources, Data curation.

## Declaration of generative AI and AI-assisted technologies in the writing process

During the preparation of this work, the author used Paperpal (https://paperpal.com) to assist with grammar and readability only and not for generating the text. After using these tools, all the authors reviewed and edited the content as needed and take full responsibility for the content of the publication.

## Funding

The authors acknowledge financial support by grants from Japan Society for the Promotion of Science (JSPS KAKENHI: JP23KJ1733 and JP24K10840). The funders had no role in study design, data collection, and analysis.

## Declaration of competing interest

The authors declare that they have no known competing financial interests or personal relationships that could have appeared to influence the work reported in this paper.
