# Supplementary material for: Computed tomography-based prediction of early recurrence risks with estimating individual times to recurrence for lung cancer patients prior to radiotherapy
Source: Phys Imaging Radiat Oncol. 2026 Jun 13;40:101021. doi: 10.1016/j.phro.2026.101021 (PMC13324663; doi:10.1016/j.phro.2026.101021)
Supplement: Supplementary file 1 — Supplementary material. [file mmc1.pdf]

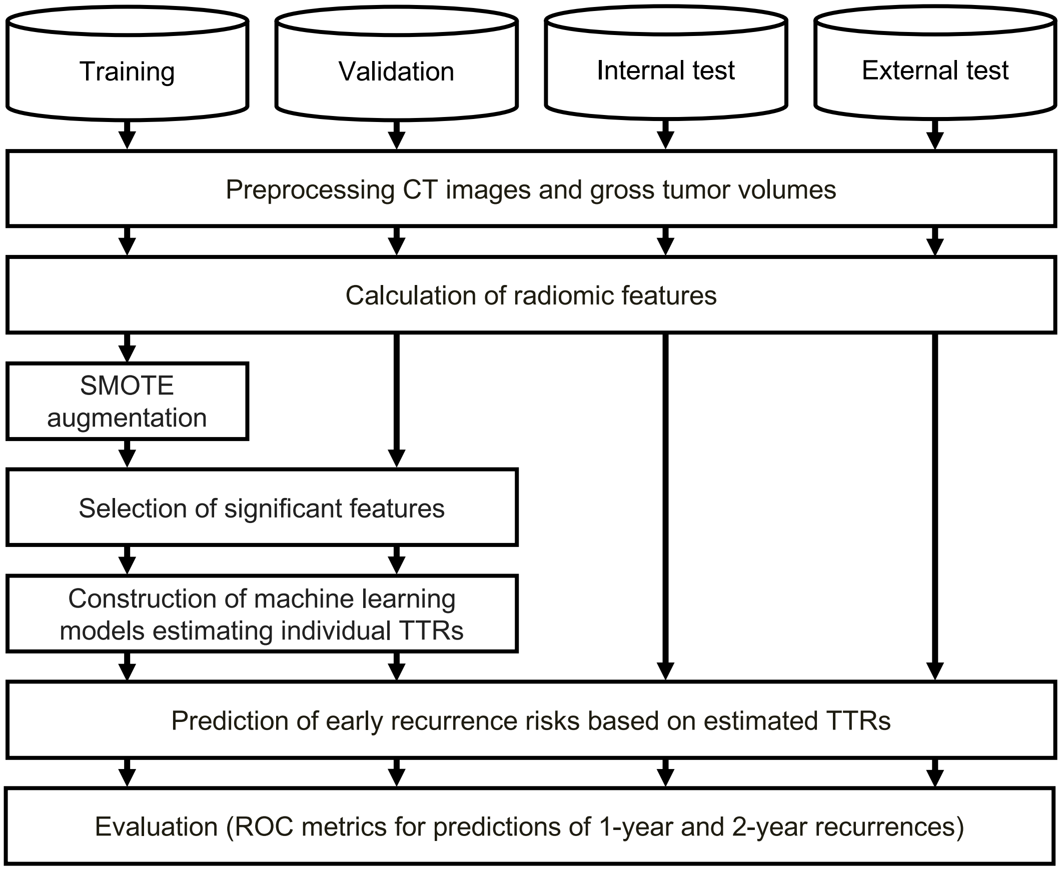

Fig. S1: Workflow of predicting early recurrence risks by estimating individual times to recurrence (TTRs). n: number of patients, CT: computed tomography, SMOTE: synthetic minority over-sampling technique, ROC: receiver operating characteristic.

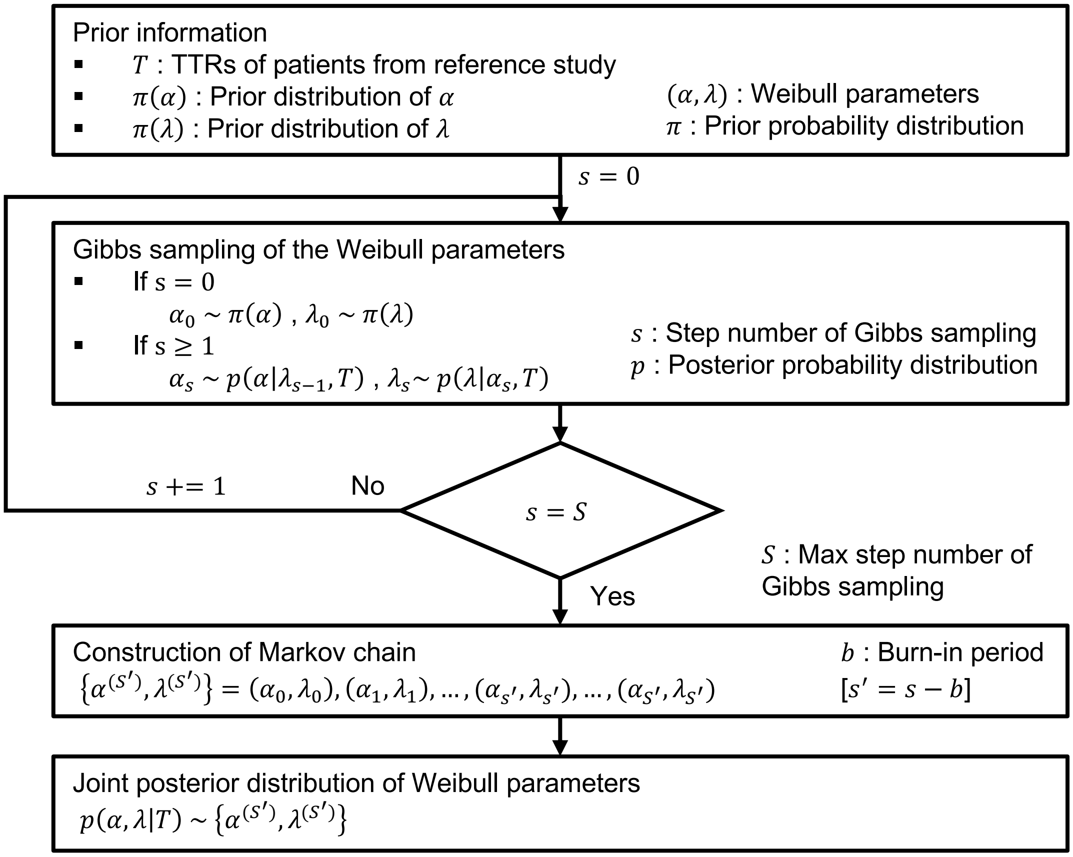

Fig. S2: Algorithm of Gibbs sampling to obtain estimated distribution of Weibull parameters. TTR: time to recurrence.

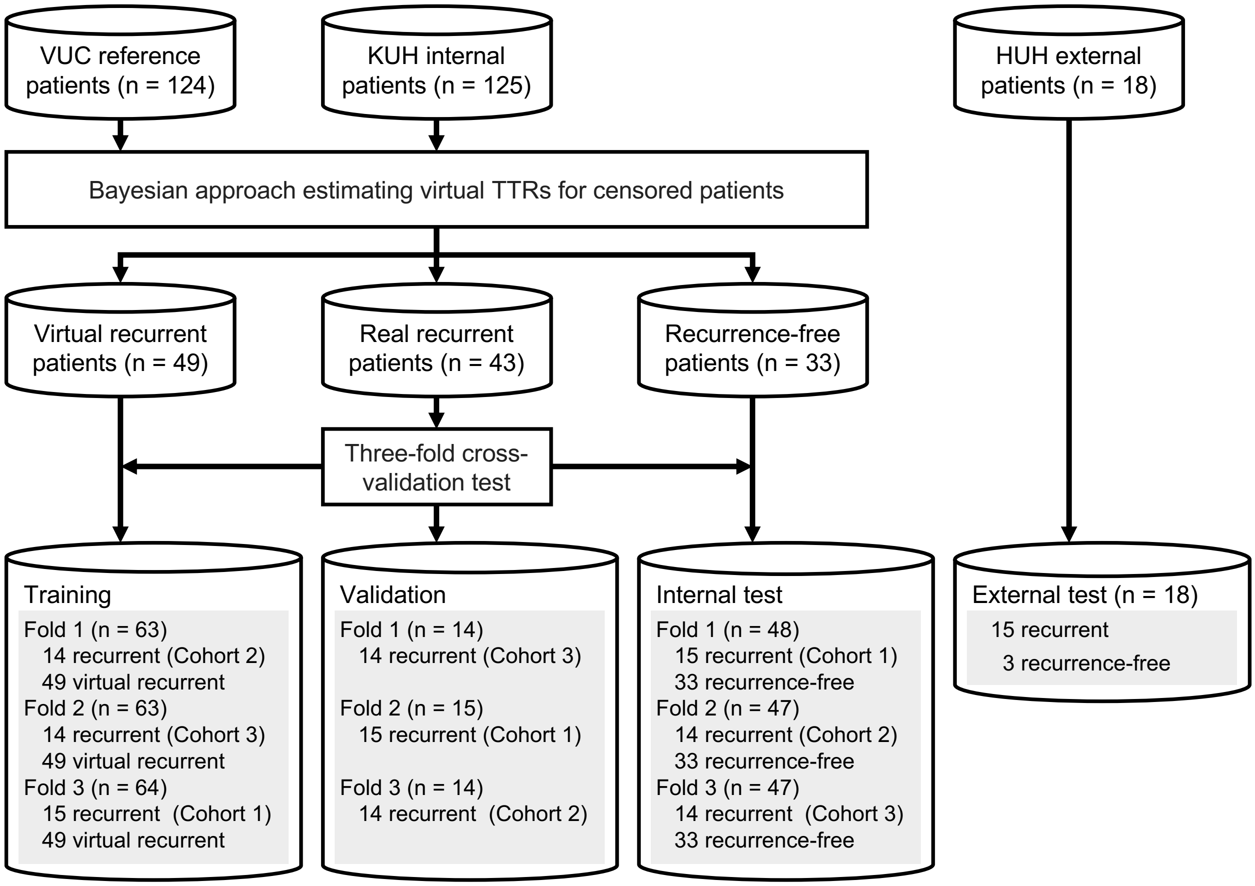

Fig. S3: Data splitting to construct training, validation, internal test, and external test datasets for the early recurrence prediction with Bayesian approach. n: number of patients, VUC: VU University Medical Center, KUH: Kyushu University Hospital, HUH: Hacettepe University Hospital. TTR: time to recurrence.

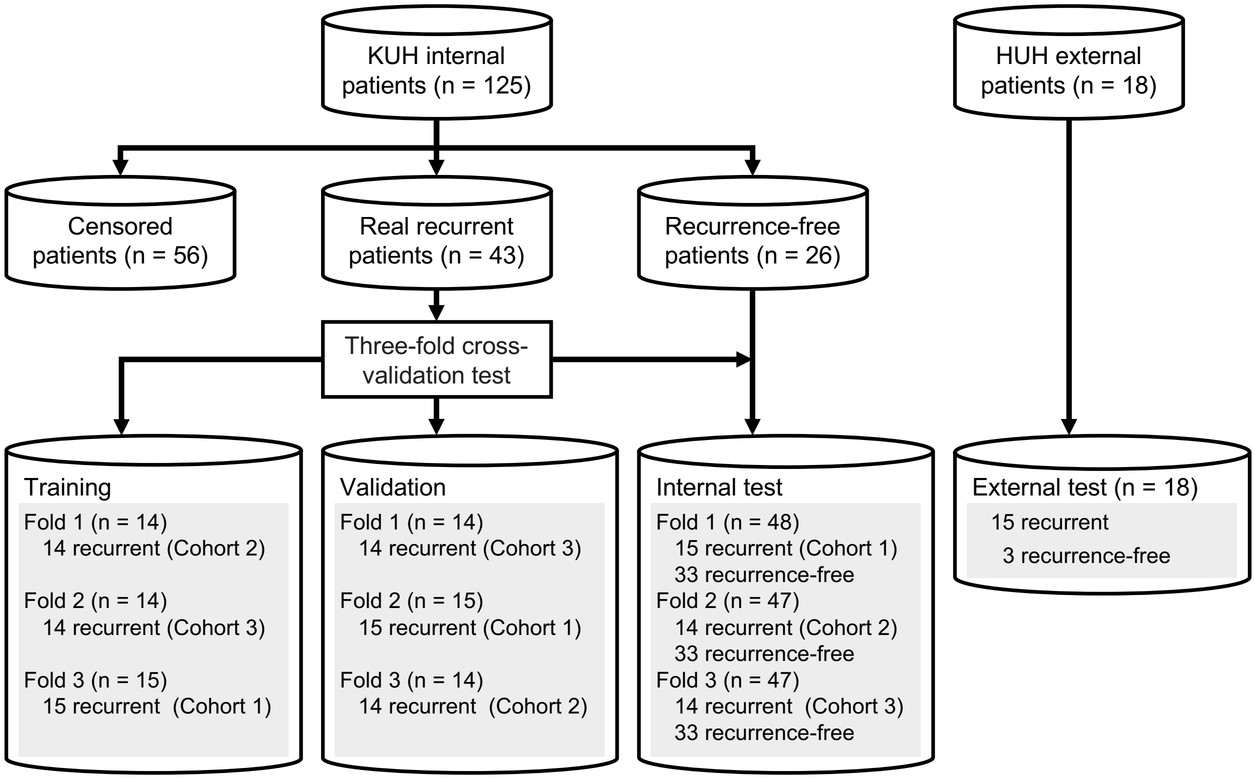

Fig. S4: Data splitting to construct training, validation, internal test, and external test datasets for the early recurrence prediction without Bayesian approach. n: number of patients, KUH: Kyushu University Hospital, HUH: Hacettepe University Hospital.

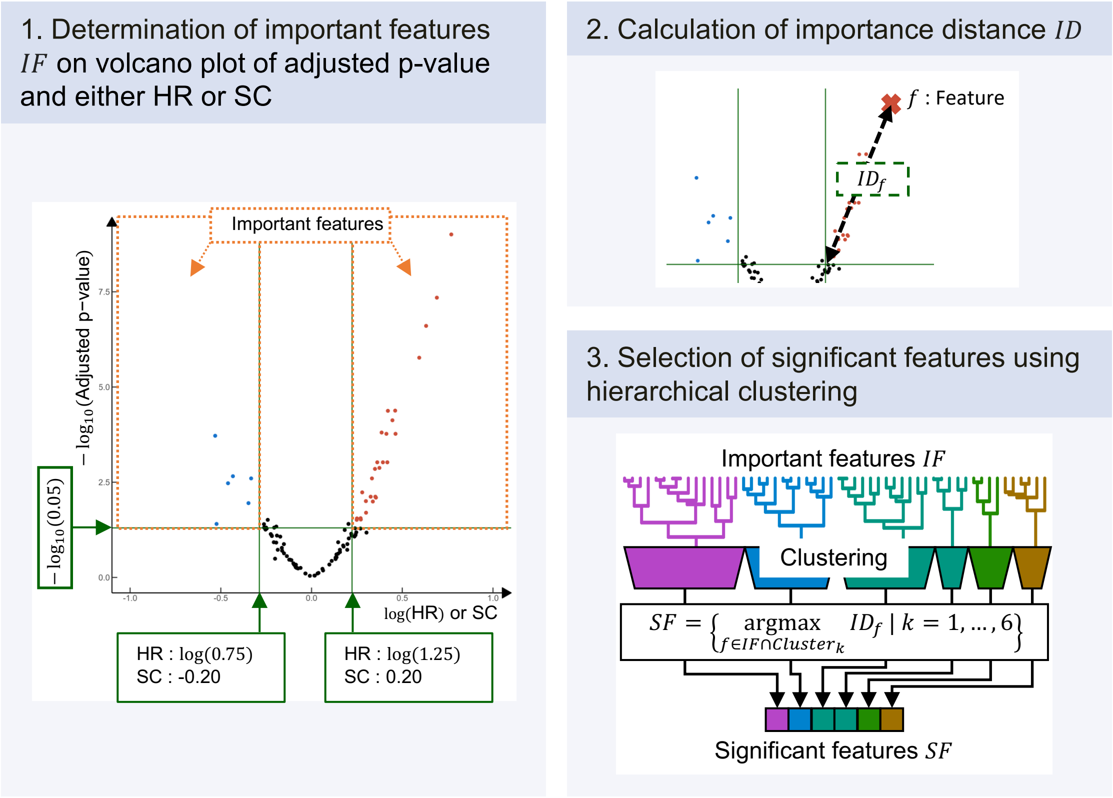

Fig. S5: Workflow of selecting significant features using Cox hazard ratio (HR) and Spearman correlation coefficient (SC) based selection methods.

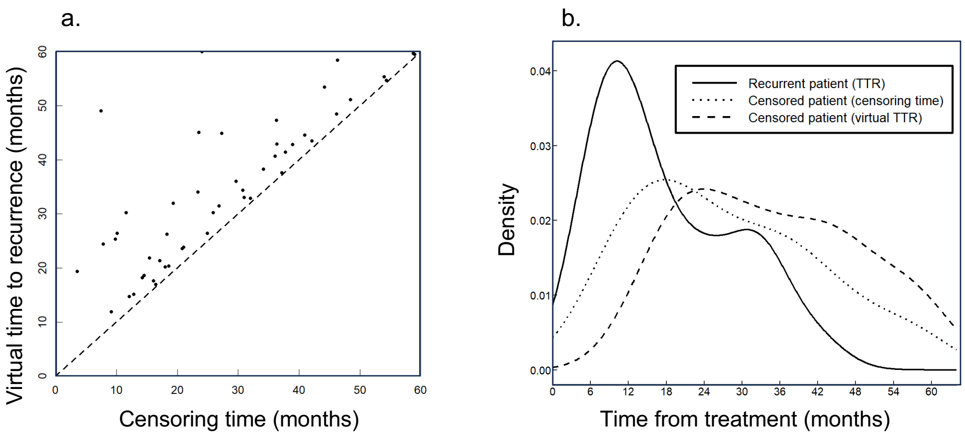

Fig. S6: (a) Scatter plot of censoring time and estimated virtual time to recurrence (TTR) for censored patients obtained from Bayesian approach. (b) Comparison of distributions of real TTR of recurrent patients, censoring time of censored patients, and virtual TTR of censored patients.

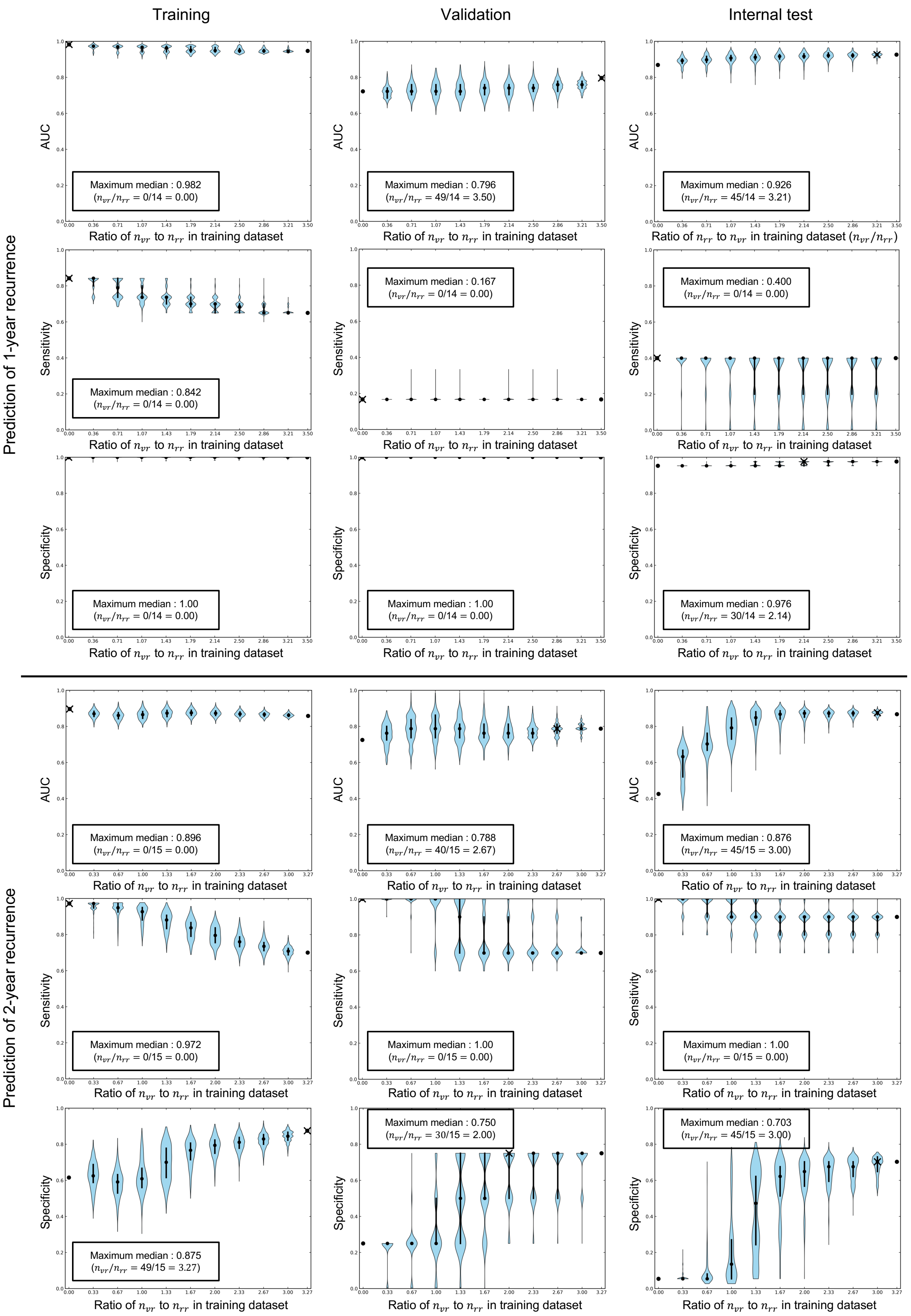

Training
Validation
Internal test
Prediction of 1-year recurrence
Prediction of 2-year recurrence
AUC
Sensitivity
Specificity
Maximum median : 0.982 (n_{vr}/n_{rr} = 0/14 = 0.00)
Maximum median : 0.796 (n_{vr}/n_{rr} = 49/14 = 3.50)
Maximum median : 0.926 (n_{vr}/n_{rr} = 45/14 = 3.21)
Maximum median : 0.842 (n_{vr}/n_{rr} = 0/14 = 0.00)
Maximum median : 0.167 (n_{vr}/n_{rr} = 0/14 = 0.00)
Maximum median : 0.400 (n_{vr}/n_{rr} = 0/14 = 0.00)
Maximum median : 1.00 (n_{vr}/n_{rr} = 0/14 = 0.00)
Maximum median : 1.00 (n_{vr}/n_{rr} = 0/14 = 0.00)
Maximum median : 0.976 (n_{vr}/n_{rr} = 30/14 = 2.14)
Maximum median : 0.896 (n_{vr}/n_{rr} = 0/15 = 0.00)
Maximum median : 0.788 (n_{vr}/n_{rr} = 40/15 = 2.67)
Maximum median : 0.876 (n_{vr}/n_{rr} = 45/15 = 3.00)
Maximum median : 0.972 (n_{vr}/n_{rr} = 0/15 = 0.00)
Maximum median : 1.00 (n_{vr}/n_{rr} = 0/15 = 0.00)
Maximum median : 1.00 (n_{vr}/n_{rr} = 0/15 = 0.00)
Maximum median : 0.875 (n_{vr}/n_{rr} = 49/15 = 3.27)
Maximum median : 0.750 (n_{vr}/n_{rr} = 30/15 = 2.00)
Maximum median : 0.703 (n_{vr}/n_{rr} = 45/15 = 3.00)
Ratio of n_{vr} to n_{rr} in training dataset
Ratio of n_{rr} to n_{vr} in training dataset (n_{vr}/n_{rr})

Fig. S7: Sensitivity analysis of the best model performance for 1-year (upper) and 2-year (lower) recurrence predictions across varying ratio of the number of virtual recurrent patients (n_{vr}) to that of real recurrent patients (n_{rr}) in the training dataset. Violin plots show the distributions of area under the receiver operating characteristic curve (AUC), sensitivity, and specificity across 1000 randomly sampled combinations for each n_{vr}. The cross mark indicates the maximum median metric.

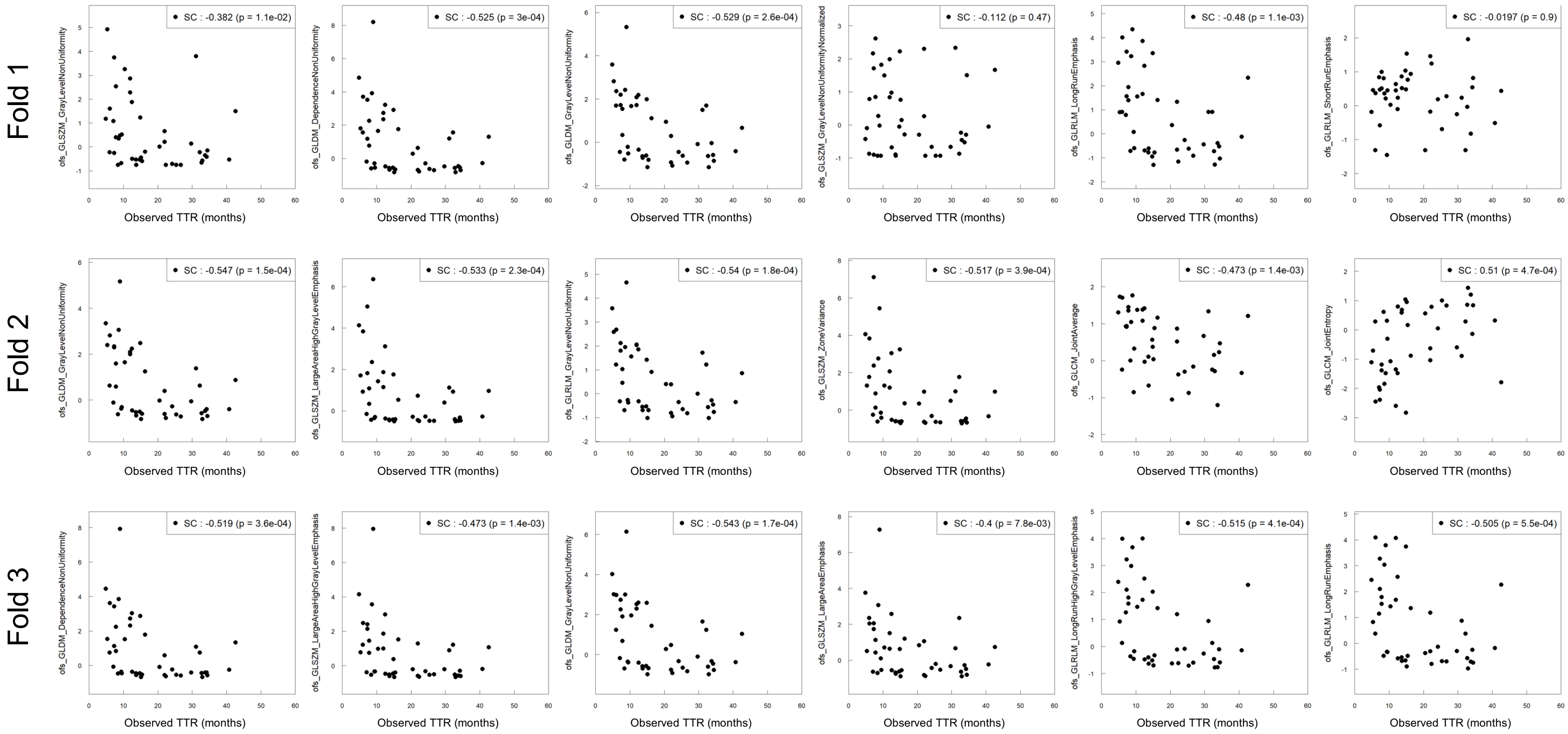

Fig. S8: Scatter plots and Spearman correlation coefficients (SC) between significant features used in the best prediction models and observed times to recurrence (TTRs) of real recurrent patients in the internal dataset

Table S1: Dose prescription of stereotactic ablative radiotherapy in the Kyushu University Hospital (KUH), Hacettepe University Hospital (HUH), and VU University Medical Center (VUC). The VUC dataset includes all patients, of whom 124 recurrent patients were used as the reference TTR distribution. BED_{10}: biologically effective dose with an α/β ratio of 10 Gy.

KUH [Gy/Fraction (BED_{10})]	n = 125	HUH [Gy/Fraction (BED10)]	n = 18	VUC [Gy / Fraction (BED10)]	n = 676
40 / 4 (80.0 Gy)	1	50 / 4 (112.5 Gy)	5	54 - 60 / 3 (151.2 - 180.0 Gy)	228
45 / 4 (95.6 Gy)	1	50 / 5 (100.0 Gy)	11	55 - 60 / 5 (115.5 - 132.0 Gy)	296
48 / 4 (105.6 Gy)	113	60 / 3 (180.0 Gy)	1	60 / 8 (105 Gy)	152
52 / 4 (119.6 Gy)	3	60 / 8 (105 Gy)	1		
54 / 4 (126.9 Gy)	3	54 / 4 (126.9 Gy)	1		
60 / 10 (96.0 Gy)	3				
70 / 10 (119.0 Gy)	1				

Table S2: Three equivalent cohorts of real recurrent patients used in a three-fold cross-validation test. n: number of patients.

	Cohort 1 (n = 15)	Cohort 2 (n = 14)	Cohort 3 (n = 14)	p-value
Age [year, min - max (median)]	70 - 91 (78)	60 - 89 (78)	60 - 86 (75.5)	> .05 (U-test)
Sex				
Male	14	12	11	> .05 (Chi-squared test)
Female	1	2	3	
Stage (ver.8)				
0	0	0	0	
IA1 / IA2 / IA3 / IB	0 / 2 / 5 / 5	0 / 3 / 5 / 3	0 / 2 / 5 / 2	> .05 (Chi-squared test)
IIA / IIB	3 / 0	3 / 0	4 / 1	
Histopathology				
Adenocarcinoma	8	7	7	
Squamous cell carcinoma	7	5	6	> .05 (Chi-squared test)
Large cell carcinoma	0	1	1	
Unknown	0	1	0	
Time to recurrence [month, min - max (median)]	4 - 42 (14)	5 - 40 (14)	5 - 34 (13.5)	> .05 (U-test)

Table S3: Event and event-free patients of 1-year and 2-year recurrences in the augmented training, validation, internal test and external test datasets for the early recurrence prediction with Bayesian approach. The external test dataset was the same across all folds.

Augmented training	Fold 1 (n = 98)	Fold 2 (n = 98)	Fold 3 (n = 98)
1-year recurrence			
Event	15	20	22
Event-free	83	78	76
2-year recurrence			
Event	50	51	50
Event-free	48	47	48
Validation	Fold 1 (n = 14)	Fold 2 (n = 15)	Fold 3 (n = 14)
1-year recurrence			
Event	6	6	5
Event-free	8	9	9
2-year recurrence			
Event	10	10	10
Event-free	4	5	4
Internal test	Fold 1 (n = 48)	Fold 2 (n = 47)	Fold 3 (n = 47)
1-year recurrence			
Event	6	5	6
Event-free	42	42	41
2-year recurrence			
Event	10	10	10
Event-free	38	37	37
External test	Fold 1 (n = 18)	Fold 2 (n = 18)	Fold 3 (n = 18)
1-year recurrence			
Event	8	8	8
Event-free	10	10	10
2-year recurrence			
Event	11	11	11
Event-free	7	7	7

Table S4: Image information of pretreatment planning computed tomography images of the Kyushu University Hospital (KUH) and Hacettepe University Hospital (HUH) patients. n: number of patients.

	KUH (n = 125)	HUH (n = 18)
Pixel size on axial plane [mm, min - max (median)]	0.781 - 0.977 (0.976)	0.973 - 1.37 (1.07)
Slice thickness [mm, min - max (median)]	2 - 5 (2)	2 - 2 (2)
Manufacturer		
Toshiba	15	18
Philips	110	0

Table S5: Calculation methods of histogram, texture, and three-dimensional shape features. The number in round brackets shows the number of features for sub-categories. GLCM: gray-level co-occurrence matrix, GLDM: gray-level dependence matrix, GLRLM: gray-level run length matrix, GLSZM: gray-level size zone matrix, NGTDM: neighboring gray tone difference matrix.

Histogram features (18)	Texture features (75)					3D shape-based features (14)
	GLCM (24)	GLDM (14)	GLRLM (16)	GLSZM (16)	NGTDM (5)	
Energy	Autocorrelation	Small Dependence Emphasis	Short Run Emphasis	Small Area Emphasis	Coarseness	Mesh Volume
Total Energy	Joint Average	Large Dependence Emphasis	Long Run Emphasis	Large Area Emphasis	Contrast	Voxel Volume
Entropy	Cluster Prominence	Gray Level Non-Uniformity	Gray Level Nonuniformity	Gray Level Non-Uniformity	Busyness	Surface Area
Minimum	Cluster Shade	Dependence Non-Uniformity	Gray Level Nonuniformity Normalized	Gray Level Non-Uniformity Normalized	Complexity	Surface Volume Ratio
10Percentile	Cluster Tendency	Dependence Non-Uniformity Normalized	Run Length Nonuniformity	Size-Zone Non-Uniformity	Strength	Sphericity
90Percentile	Contrast	Gray Level Variance	Run Length Nonuniformity Normalized	Size-Zone Non-Uniformity Normalized		Compactness 1
Maximum	Correlation	Dependence Variance	Run Percentage	Zone Percentage		Compactness 2
Mean	Difference Average	Dependence Entropy	Gray Level Variance	Gray Level Variance		Spherical Disproportion
Median	Difference Entropy	Low Gray Level Emphasis	Run Variance	Zone Variance		Maximum 3D Diameter
Interquartile Range	Difference Variance	High Gray Level Emphasis	Run Entropy	Zone Entropy		Major Axis Length
Range	Joint Energy	Small Dependence Low Gray Level Emphasis	Low Gray Level Run Emphasis	Low Gray Level Zone Emphasis		Minor Axis Length
Mean Absolute Deviation	Joint Entropy	Small Dependence High Gray Level Emphasis	High Gray Level Run Emphasis	High Gray Level Zone Emphasis		Least Axis Length
Robust Mean Absolute Deviation	Informational Measure of Correlation1(Imc1)	Large Dependence Low Gray Level Emphasis	Short Run Low Gray Level Emphasis	Small Area Low Gray Level Emphasis		Elongation

Root Mean Squared	Informational Measure of Correlation 2 (Imc2)	Large Dependence High Gray Level Emphasis	Level Emphasis	Small Area High Gray Level Emphasis		Flatness
Skewness	Inverse Difference Moment		Long Run Low Gray Level Emphasis	Large Area Low Gray Level Emphasis		
Kurtosis	Maximal Correlation Coefficient		Long Run High Gray Level Emphasis	Large Area High Gray Level Emphasis		
Variance	Inverse Difference Moment Normalized					
Uniformity	Inverse Difference					
	Inverse Difference Moment Normalized					
	Inverse Variance					
	Maximum Probability					
	Sum Average					
	Sum Entropy					
	Sum Squares					

Table S6: Hyperparameters of regression models

Model	Parameter	Description	Range	Data type
Elastic Net [1]	alpha	Constant that multiplies (weights) the penalty terms	[1e-4, 1e4]	Float
	l1_ratio	ElasticNet mixing parameter of a combination of L1 and L2 penalties	[0, 1]	Float
Random Forest Regressor [1]	n_estimators	Number of decision trees	[10, 1000]	Integer
	max_depth	Maximum depth of the decision tree	[5, 30]	Integer
	min_samples_split	Minimum number of samples required to split an internal node	[2, 20]	Integer
	min_samples_leaf	Minimum number of samples required to be at a leaf node	[1, 10]	Integer
XGBRegressor [2]	subsample	Subsample ratio of the training instance	[0, 1]	Float
	colsample_bytree	Subsample ratio of columns when constructing each tree	[0, 1]	Float
	reg_alpha	L1 regularization term on weights	[0, 10]	Float
	reg_lambda	L2 regularization term on weights	[0, 10]	Float
	learning_rate	Boosting learning rate	[1e-5, 1]	Float
	min_child_weight	Minimum sum of instance weight needed in a child leaf	[1, 15]	Integer
	max_depth	Maximum tree depth for base learners	[1, 11]	Integer
	gamma	Minimum loss reduction required to make a further partition on a leaf node of the tree	[1e-5, 10]	Float
Cox regression [3]	penalizer	Constant that coefficients (weights) of the penalty terms	[0, 10]	Float
	l1_ratio	ElasticNet mixing parameter of a combination of L1 and L2 penalties	[0, 1]	Float
Random Survival Forest [4]	n_estimators	Number of decision trees	[10, 1000]	Integer
	max_depth	Maximum depth of the decision tree	[5, 30]	Integer
	min_samples_split	Minimum number of samples required to split an internal node	[2, 20]	Integer
	min_samples_leaf	Minimum number of samples required to be at a leaf node	[1, 10]	Integer

[1] scikit-learn. https://scikit-learn.org/stable/
[2] XGBoost Documentation. https://xgboost.readthedocs.io/en/stable/
[3] Lifelines Ver. 0.30.3. https://lifelines.readthedocs.io/en/latest/index.html
[4] Scikit-survival Ver. 0.27.0. https://scikit-survival.readthedocs.io/en/stable/index.html

TableS7: Comparison of baseline characteristics of recurrent, recurrence-free, and censored patients in the Kyushu University Hospital

	Recurrent (R; n = 43)	Recurrence-free (RF; n = 26)	Censored (C; n = 56)	Statistical difference
Age [year, min - max (median)]	60 - 91 (78)	64 - 89 (74.5)	60 - 89 (79)	Mann-Whitney U test<br>R vs. RF: $p = 0.264$,<br>R vs. C: $p = 0.149$,<br>C vs. RF: $p = 1.00\times10^{-2}$
Sex				Chi-squared test<br>R vs. RF: $p = 8.08\times10^{-3}$,
Male	37	15	38	R vs. C: $p = 3.63\times10^{-2}$,
Female	6	11	18	C vs. RF: $p = 0.370$
Stage (UICC 8th edition)				Chi-squared test
0	0	8	2	R vs. RF: $p = 2.73\times10^{-4}$,
IA1 / IA2 / IA3 / IB	0 / 7 / 15 / 10	2 / 5 / 9 / 2	1 / 22 / 17 / 10	R vs. C: $p = 4.35\times10^{-2}$,
IIA / IIB	10 / 1	0 / 0	4 / 0	C vs. RF: $p = 3.15\times10^{-3}$
Histopathology				Chi-squared test
Adenocarcinoma	22	19	33	R vs. RF: $p = 0.232$,
Squamous cell carcinoma	18	6	18	R vs. C: $p = 0.678$,
Large cell carcinoma	2	0	2	C vs. RF: $p = 0.558$
Unknown	1	1	3	
Dose prescription				Chi-squared test
40 Gy / 4 fractions	0	0	1	R vs. RF: $p = 0.536$,
45 Gy / 4 fractions	0	0	1	R vs. C: $p = 0.501$,
48 Gy / 4 fractions	41	26	46	C vs. RF: $p = 0.507$
52 Gy / 4 fractions	1	0	2	
54 Gy / 4 fractions	1	0	2	
60 Gy / 10 fractions	0	0	3	
70 Gy / 10 fractions	0	0	1	

Table S8: Receiver operating characteristic (ROC) metrics of 1-year recurrence prediction in a three-fold cross-validation. HR: Cox hazard ratio, SC: Spearman correlation coefficient, EN: elastic net, RF: random forest, CR: Cox regression, RSF: random survival forest, AUC: area under the ROC curve.

Bayesian approach	Feature	Selection – estimation models	Fold	Training AUC	Validation AUC	Internal test AUC	Accuracy	Sensitivity	Specificity
w/	Original	HR - RF	1	0.943	0.750	0.766	0.917	0.333	1.000
			2	0.947	0.796	0.926	0.915	0.400	0.976
			3	0.940	0.878	0.860	0.894	0.333	0.976
			Mean	0.943	0.808	0.851	0.908	0.356	0.984
	Wavelet	HR - RF	1	0.972	0.708	0.937	0.917	0.333	1.000
			2	0.938	0.852	0.783	0.936	0.400	1.000
			3	0.959	0.900	0.797	0.894	0.333	0.976
			Mean	0.956	0.820	0.839	0.915	0.356	0.992
	Betti number map	HR - RF	1	0.918	0.792	0.750	0.917	0.333	1.000
			2	0.990	0.741	0.881	0.915	0.400	0.976
			3	0.904	0.833	0.793	0.894	0.333	0.976
			Mean	0.937	0.789	0.808	0.908	0.356	0.984
w/o	Original	SC - EN	1	0.867	0.813	0.710	0.896	0.500	0.952
			2	0.935	0.731	0.933	0.915	0.600	0.952
			3	0.861	0.811	0.793	0.787	0.333	0.854
			Mean	0.888	0.785	0.812	0.866	0.478	0.919
	Wavelet	HR - EN	1	0.933	0.813	0.742	0.938	0.667	0.976
			2	0.948	0.741	0.950	0.915	0.800	0.929
			3	0.838	0.878	0.705	0.894	0.333	0.976
			Mean	0.906	0.810	0.799	0.915	0.600	0.960
	Betti number map	HR - RF	1	0.952	0.813	0.631	0.750	0.667	0.762
			2	1.000	0.704	0.860	0.957	0.600	1.000
			3	0.991	0.878	0.850	0.702	0.667	0.707
			Mean	0.981	0.798	0.780	0.803	0.644	0.823
	Original	HR - CR	1	0.799	0.813	0.667	0.400	1.00	0.00
			2	0.779	0.796	0.900	0.571	1.00	0.333
			3	0.787	0.900	0.667	0.429	1.00	0.00
			Mean	0.788	0.836	0.744	0.467	1.00	0.111
	Original	HR - RSF	1	0.831	0.771	0.685	0.400	1.00	0.00
			2	0.858	0.870	0.944	0.714	1.00	0.556
			3	0.885	0.900	0.646	0.571	1.00	0.250
			Mean	0.858	0.847	0.758	0.562	1.00	0.269

Table S9: Receiver operating characteristic (ROC) metrics of 2-year recurrence prediction in a three-fold cross-validation. HR: Cox hazard ratio, SC: Spearman correlation coefficient, EN: elastic net, RF: random forest, CR: Cox regression, RSF: random survival forest, AUC: area under the ROC curve.

Bayesian approach	Feature	Selection – estimation models	Fold	Training AUC	Validation AUC	Internal test AUC	Accuracy	Sensitivity	Specificity
w/	Original	HR - RF	1	0.887	0.700	0.703	0.583	0.600	0.579
			2	0.875	0.680	0.834	0.766	0.600	0.811
			3	0.858	0.788	0.869	0.745	0.900	0.703
			Mean	0.873	0.723	0.802	0.698	0.700	0.697
	Wavelet	HR - RF	1	0.880	0.725	0.708	0.792	0.500	0.868
			2	0.945	0.860	0.769	0.745	0.500	0.811
			3	0.876	0.813	0.757	0.766	0.600	0.811
			Mean	0.900	0.799	0.745	0.767	0.533	0.830
	Betti number map	HR - RF	1	0.911	1.000	0.762	0.667	0.700	0.658
			2	0.931	0.940	0.697	0.723	0.600	0.757
			3	0.896	0.763	0.799	0.787	0.500	0.865
			Mean	0.912	0.901	0.753	0.726	0.600	0.760
w/o	Original	SC - EN	1	0.884	0.825	0.658	0.208	0.800	0.053
			2	0.896	0.690	0.711	0.426	0.700	0.351
			3	0.835	0.537	0.832	0.468	1.000	0.324
			Mean	0.872	0.684	0.734	0.367	0.833	0.243
	Wavelet	HR - EN	1	0.806	0.825	0.697	0.313	1.000	0.132
			2	0.905	0.640	0.759	0.213	1.000	0.000
			3	0.751	0.563	0.761	0.255	1.000	0.054
			Mean	0.821	0.676	0.739	0.260	1.000	0.062
	Betti number map	HR - RF	1	0.891	0.850	0.733	0.271	0.900	0.105
			2	0.986	0.900	0.623	0.617	0.500	0.649
			3	0.917	0.688	0.697	0.426	0.900	0.297
			Mean	0.931	0.813	0.684	0.438	0.767	0.350
	Original	HR - CR	1	0.757	0.875	0.560	0.667	1.00	0.00
			2	0.714	0.740	0.863	0.714	1.00	0.00
			3	0.784	0.938	0.550	0.714	1.00	0.00
			Mean	0.752	0.851	0.657	0.698	1.00	0.00
	Original	HR - RSF	1	0.776	0.775	0.720	0.667	1.00	0.00
			2	0.768	0.760	0.813	0.714	1.00	0.00
			3	0.858	0.888	0.850	0.714	1.00	0.00
			Mean	0.801	0.808	0.794	0.698	1.00	0.00

Table S10: Receiver operating characteristic (ROC) metrics of early recurrence predictions of the best prediction models in the internal and external tests. HR: Cox hazard ratio, RF: random forest, AUC: area under the ROC curve.

					Internal test				External test			
Recurrence	Bayesian approach	Feature	Selection - estimation models	Fold	AUC	Accuracy	Sensitivity	Specificity	AUC	Accuracy	Sensitivity	Specificity
1-year recurrence	w/	Original	HR - RF	2	0.926	0.915	0.400	0.976	0.763	0.611	0.125	1.000
2-year recurrence	w/	Original	HR - RF	3	0.869	0.745	0.900	0.703	0.883	0.778	0.727	0.857

Table S11: Concordance correlation coefficients in the three-fold cross-validation for real recurrent patients. HR: Cox hazard ratio, SC: Spearman correlation coefficient, RF: random forest, EN: elastic-net.

Bayesian approach	Feature	Selection – estimation models	Fold	Training	Validation	Internal test
w/	Original	HR - RF	1	0.672	0.347	0.282
			2	0.582	0.260	0.501
			3	0.591	0.590	0.536
			Mean	0.615	0.399	**0.440**
	Wavelet	HR - RF	1	0.604	0.345	0.335
			2	0.743	0.525	0.383
			3	0.598	0.590	0.370
			Mean	0.648	0.487	0.363
	Betti number map	HR - RF	1	0.684	0.679	0.477
			2	0.744	0.684	0.394
			3	0.627	0.525	0.428
			Mean	0.685	0.629	0.433
w/o	Original	SC - EN	1	0.527	0.551	0.030
			2	0.653	0.124	0.374
			3	0.362	0.365	0.468
			Mean	0.514	0.347	0.291
	Wavelet	HR - EN	1	0.594	0.592	0.129
			2	0.589	0.231	0.428
			3	0.286	0.313	0.314
			Mean	0.490	0.379	0.290
	Betti number map	HR - RF	1	0.774	0.636	0.150
			2	0.947	0.509	0.418
			3	0.818	0.540	0.225
			Mean	0.846	0.561	0.264

Table S12: Concordance index (c-index) and p-values (log-rank test) of Kaplan-Meier curves between observed and estimated times to recurrence in a three-fold cross-validation test for real recurrent patients. CI: confidence interval, HR: Cox hazard ratio, SC: Spearman correlation coefficient, RF: random forest, EN: elastic-net.

				Training		Validation		Internal test	
Bayesian approach	Feature	Selection - estimation models	Fold	p-value	c-index (95% CI)	p-value	c-index (95% CI)	p-value	c-index (95% CI)
w/	Original	HR - RF	1	0.774	0.781 (0.66 - 0.90)	0.521	0.673 (0.53 - 0.74)	0.924	0.968 (0.94 - 1.00)
			2	0.759	0.758 (0.66 - 0.85)	0.900	0.657 (0.48 - 0.83)	0.955	0.971 (0.94 - 1.00)
			3	0.621	0.841 (0.73 - 0.95)	0.990	0.720 (0.61 - 0.83)	0.962	0.971 (0.94 - 1.00)
			Mean	-	0.793	-	0.671	-	0.970
	Wavelet	HR - RF	1	0.999	0.795 (0.68 - 0.91)	0.725	0.615 (0.47 - 0.76)	0.930	0.973 (0.94 - 1.00)
			2	0.923	0.780 (0.66 - 0.90)	0.600	0.733 (0.62 - 0.84)	0.935	0.971 (0.94 - 1.00)
			3	0.921	0.868 (0.78 - 0.96)	0.940	0.753 (0.64 - 0.86)	0.965	0.972 (0.94 - 1.00)
			Mean	-	0.815	-	0.700	-	0.972
	Betti number map	HR - RF	1	0.903	0.748 (0.62 - 0.88)	0.449	0.758 (0.62 - 0.89)	0.935	0.974 (0.95 - 1.00)
			2	0.310	0.874 (0.78 - 0.97)	0.609	0.724 (0.58 - 0.86)	0.930	0.975 (0.95 - 1.00)
			3	0.928	0.747 (0.64 - 0.85)	0.610	0.709 (0.54 - 0.87)	0.924	0.971 (0.94 - 1.00)
			Mean	-	0.789	-	0.730	-	0.973
w/o	Original	SC - EN	1	0.277	0.752 (0.65 - 0.86)	0.369	0.703 (0.57 - 0.83)	0.992	0.956 (0.91 - 1.00)
			2	0.439	0.758 (0.65 - 0.87)	0.241	0.633 (0.43 - 0.83)	0.998	0.970 (0.94 - 1.00)
			3	0.467	0.758 (0.57 - 0.94)	0.655	0.643 (0.46 - 0.82)	0.988	0.968 (0.93 - 1.00)
			Mean	-	0.756	-	0.660	-	0.964
	Wavelet	HR - EN	1	0.168	0.710 (0.55 - 0.87)	0.363	0.736 (0.61 - 0.86)	1.00	0.961 (0.92 - 1.00)
			2	0.382	0.786 (0.66 - 0.91)	0.178	0.657 (0.47 - 0.84)	0.959	0.975 (0.95 - 1.00)
			3	0.369	0.747 (0.56 - 0.94)	0.169	0.610 (0.45 - 0.77)	0.999	0.969 (0.94 - 1.00)
			Mean	-	0.747	-	0.668	-	0.968

Betti number map	HR - RF	1	0.489	0.767 (0.63 - 0.91)	0.509	0.692 (0.57 - 0.81)	0.988	0.965 (0.93 - 1.00)
		2	0.448	0.923 (0.85 - 1.0)	0.511	0.686 (0.54 - 0.83)	0.962	0.970 (0.94 - 1.00)
		3	0.472	0.824 (0.74 - 0.91)	0.876	0.736 (0.60 - 0.87)	0.987	0.967 (0.93 - 1.00)
		Mean	-	0.838	-	0.705	-	0.967

Table S13: Optimal sets of hyperparameters of estimation models and significant features

Prediction model				Image parameters								
Features	Bayesian approach	Selection - estimation models	Fold	Requantization window	Requantization bit depth	Kernel size / shift pixel (for Betti number map)	Significant features					
Original (OFs)	w/	HR-RF	1	Full	7	-	OFs_GLSZM_GrayLevelNonUniformity	OFs_GLDM_DependenceNonUniformity	OFs_GLDM_GrayLevelNonUniformity	OFs_GLSZM_GrayLevelNonUniformityNormalized	OFs_GLRLM_LongRunEmphasis	OFs_GLRLM_ShortRunEmphasis
			2	Lung	9	-	OFs_GLDM_GrayLevelNonUniformity	OFs_GLSZM_LargeAreaHighGrayLevelEmphasis	OFs_GLRLM_GrayLevelNonUniformity	OFs_GLSZM_ZoneVariance	OFs_GLCM_JointAverage	OFs_GLCM_JointEntropy
			3	Full	8	-	OFs_GLDM_DependenceNonUniformity	OFs_GLSZM_LargeAreaHighGrayLevelEmphasis	OFs_GLDM_GrayLevelNonUniformity	OFs_GLSZM_LargeAreaEmphasis	OFs_GLRLM_LongRunHighGrayLevelEmphasis	OFs_GLRLM_LongRunEmphasis
Wavelet (WFs)	w/	HR - RF	1	Lung	9	-	WFs_GLRLM_RunVariance_HLL	WFs_GLDM_GrayLevelNonUniformity_LLL	WFs_GLSZM_LargeAreaHighGrayLevelEmphasis_LLL	WFs_GLDM_DependenceVariance_HLH	WFs_GLRLM_LongRunHighGrayLevelEmphasis_LHH	WFs_GLDM_LargeDependenceEmphasis_LHL
			2	Lung	9	-	WFs_GLDM_GrayLevelNonUniformity_LLL	WFs_GLSZM_ZoneVariance_LLL	WFs_GLDM_DependenceVariance_HLL	WFs_GLRLM_LongRunEmphasis_HLL	WFs_GLCM_JointEnergy_LHL	WFs_FirstOrder_Energy_LLH
			3	Mediastinal	8	-	WFs_GLSZM_LargeAreaHighGrayLevelEmphasis_LLL	WFs_GLSZM_SizeZoneNonUniformity_LHL	WFs_NGTDM_Busyness_HLL	WFs_GLRLM_RunLengthNonUniformity_LHL	WFs_NGTDM_Busyness_HLH	WFs_GLSZM_GrayLevelNonUniformity_HLL

Betti number map (BMFs)	w/	HR - RF	1	Full	8	7 / 2	BMFs_FirstOrder_Kurtosis_B0_t78	BMFs_FirstOrder_Kurtosis_B0_t80	BMFs_FirstOrder_Skewness_B0_t81	BMFs_FirstOrder_Skewness_B0_t78	BMFs_FirstOrder_Skewness_iB0_t10	BMFs_FirstOrder_Energy_B0_t94
			2	Full	8	7 / 2	BMFs_FirstOrder_Kurtosis_B0_t86	BMFs_FirstOrder_Energy_B10_t107	BMFs_FirstOrder_Skewness_B0_t81	BMFs_FirstOrder_Skewness_iB0_t11	BMFs_FirstOrder_Energy_iB0_t120	BMFs_FirstOrder_RobustMeanAbsoluteDeviation_iB0_t96
			3	Full	7	5 / 2	BMFs_FirstOrder_Kurtosis_B0_t39	BMFs_FirstOrder_Kurtosis_B0_t40	BMFs_FirstOrder_Skewness_iB0_t18	BMFs_GLDM_DependenceNonUniformity_B0_t0	BMFs_FirstOrder_Energy_iB0_t54	BMFs_FirstOrder_Kurtosis_iB0_t6
Original (OFs)	w/o	SC - EN	1	Lung	9	-	OFs_NGTDM_Contrast	OFs_FirstOrder_Kurtosis	OFs_FirstOrder_10Percentile	OFs_GLDM_LargeDependenceEmphasis	OFs_GLRLM_LowGrayLevelRunEmphasis	OFs_GLRLM_RunPercentage
			2	Lung	8	-	OFs_GLDM_LargeDependenceHighGrayLevelEmphasis	OFs_FirstOrder_90Percentile	OFs_FirstOrder_RootMeanSquared	OFs_GLRLM_LowGrayLevelRunEmphasis	OFs_GLCM_MaximumProbability	OFs_GLDM_SmallDependenceLowGrayLevelEmphasis
			3	Lung	8	-	OFs_NGTDM_Busyness	OFs_Shape_Maximum2DDiameterColumn	OFs_GLSZM_LargeAreaLowGrayLevelEmphasis	OFs_Shape_LeastAxisLength	OFs_GLSZM_LowGrayLevelZoneEmphasis	OFs_NGTDM_Coarseness
Wavelet (WFs)	w/o	HR - EN	1	Mediastinal	7	-	WFs_FirstOrder_Median_LLL	WFs_FirstOrder_Kurtosis_LHL	WFs_GLSZM_SizeZoneNonUniformity_LLH	WFs_GLSZM_SizeZoneNonUniformity_HLH	WFs_FirstOrder_Mean_LLL	WFs_FirstOrder_Mean_HHL
			2	Full	9	-	WFs_GLSZM_LargeAreaLowGrayLevelEmphasis_HHL	WFs_FirstOrder_Uniformity_HLL	WFs_GLCM_Id_HLL	WFs_GLCM_Idm_HLL	WFs_GLRLM_LongRunEmphasis_HLL	WFs_GLRLM_RunEntropy_HHL
			3	Lung	7	-	WFs_GLSZM_LargeAreaLowGrayLevelEmphasis_LLH	WFs_NGTDM_Strength_LLL	WFs_GLRLM_GrayLevelNonUniformity_LLL	WFs_GLRLM_GrayLevelNonUniformity_LLH	WFs_GLDM_DependenceNonUniformity_LLL	WFs_GLSZM_SmallAreaEmphasis_LHH

Betti number map (BMFs)	w/o	HR - RF	1	Full	8	7 / 1	BMFs_FirstOrder_Kurtosis_b0_t87	BMFs_FirstOrder_Skewness_b0_t66	BMFs_FirstOrder_Skewness_ib0_t52	BMFs_FirstOrder_Variance_ib0_t91	BMFs_FirstOrder_Energy_b0_t92	BMFs_FirstOrder_RootMeanSquared_ib0_t30
			2	Lung	9	9 / 2	BMFs_FirstOrder_Kurtosis_ib0_t346	BMFs_FirstOrder_Energy_ib1_t488	BMFs_FirstOrder_MeanAbsoluteDeviation_ib0_t420	BMFs_FirstOrder_Kurtosis_b0_t449	BMFs_FirstOrder_Skewness_ib1_t441	BMFs_FirstOrder_Skewness_b0_t411
			3	Full	9	9 / 2	BMFs_FirstOrder_Kurtosis_ib0_t169	BMFs_FirstOrder_Kurtosis_ib0_t167	BMFs_FirstOrder_Energy_ib0_t208	BMFs_FirstOrder_90Percentile_ib10_t212	BMFs_FirstOrder_Kurtosis_ib0_t23	BMFs_FirstOrder_Range_ib0_t190

Document S1: Software specifications

- Bayesian approach

The Gibbs sampling algorithm fitting a probability density distribution function to reference times to recurrence (TTRs) was performed using the "rjags" [rjags] package in R.

- Preprocessing of planning computed tomography (pCT) image and gross tumor volume (GTV)

The anisotropic pCT image and GTV were preprocessed by iso-voxelization, noise reduction using a Laplacian of Gaussian (LoG) filter, and re-quantization to q bits before computation of radiomic feature extraction. Isotropic images and GTVs were created with a voxel size of 0.977 mm using cubic and shape-based interpolation [Herman 1992], respectively. To enhance the lung cancer patterns and reduce noise, the LoG filtered image was subtracted from the isotropic image. Re-quantization rescales the filtered image intensity into q bits based on a lookup table corresponding to diagnostic windows of the Hounsfield Units (HUs). Three re-quantization bit depths of 7, 8, and 9 bits and three window settings (window levels/widths of HUs) of the full window (250 / 2500), mediastinal window (50 / 400), and lung window (-600 / 1500) [Takahashi 2019] were optimized as imaging parameters. The iso-voxelization, noise reduction, and re-quantization were performed in the MATLAB 2023a environment using in-house functions.

- Extraction of radiomic features

Three types of radiomic features were calculated from preprocessed pCT (original) images, wavelet decomposed images, and Betti number maps using the python radiomics package "Pyradiomics" [Pyradiomics]. The Betti number maps were calculated using in-house functions published in previous studies [Ninomiya 2020, Kodama 2022].

- Synthetic minority over-sampling technique (SMOTE) augmentation

SMOTE augmentation was used to maintain the original demographics of the TTRs in the training patients by using the SMOTE function in the “imbalanced-learning” package [imlearn].

- Selection of significant features

Significant features were selected using two selection methods based on Cox hazard ratio and Spearman correlation coefficient. Feature selection was performed using in-house functions in R, based on a previous study [Jin 2025]. Hierarchical clustering in the selection methods was performed using the “proxy” [proxy] package.

- Construction of TTR estimation models

Estimation of the TTRs was investigated using three types of machine learning regression models: elastic-net [sklearn], random forest [sklearn], and extreme-gradient boosting (XGB) [XGBoost]. The hyperparameters of the estimation models were optimized 100 times iterations of Bayesian optimization [Nogueira 2014].

- Evaluation

Concordance correlation coefficients, which evaluate the correlation between the observed and estimated TTRs, were computed using the “torchmetrics” [torchmetrics] package in Python. Receiver operating characteristic (ROC) metrics of area under the ROC curve, accuracy, sensitivity, and specificity were calculated to evaluate the early recurrence predictions using the “scikit-learn” package [sklearn] in Python. Uniform manifold approximation and projection (UMAP) [McInnes 2018] investigated the association between significant feature vectors and TTRs.

References;

[rjags] rjags. https://cran.r-project.org/web/packages/rjags/rjags.pdf. (accessed 12 Dec 2025).

[Herman 1992] Herman GT, Zheng J and Bucholtz CA. Shape-based interpolation. IEEE Comput Graph. 1992;12(3):69-79. https://doi.org/10.1109/38.135915.

[Takahashi 2019] Takahashi H, Kiyoshima M, Kaburagi T, Hoshiai S, Mori K, Koyama K, et al. Influence of radiologic expertise in detecting lung tumors on chest radiographs. Diagnostic and interventional imaging. 2019;100(2):95-107. https://doi.org/10.1016/j.diii.2018.08.015.

[Pyradiomics] Pyradiomics. https://pyradiomics.readthedocs.io/en/latest/. (accessed 12 Dec 2025).

[Ninomiya 2020] Ninomiya K, Arimura H. Homological radiomics analysis for prognostic prediction in lung cancer patients. Physica Medica. 2020;69:90-100. https://doi.org/10.1016/j.ejmp.2019.11.026.

[Kodama 2022] Kodama T, Arimura H, Shirakawa Y, Ninomiya K, Yoshitake T, Shioyama Y. Relapse predictability of topological signature on pretreatment planning CT images of stage I non-small cell lung cancer patients before treatment with stereotactic ablative radiotherapy. Thorac Cancer. 2022;13(15):2117-2126. https://doi.org/10.1111/1759-7714.14483.

[imlearn] imbalanced-learn. https://imbalanced-learn.org/stable/. (accessed 12 Dec 2025).

[Jin 2025] Jin Y, Arimura H, Iwasaki T, Kodama T, Yamamoto N, Cui Y, et al. Multiscale Fusion Models With Genomic, Topological, and Pathomic Features to Predict Response to Radiation Therapy for Non-Small Cell Lung Cancer Patients. Lab Invest. 2025;105(10):104204. https://doi.org/10.1016/j.labinv.2025.104204.

[proxy] proxy. https://cran.r-project.org/web/packages/proxy/proxy.pdf. (accessed 12 Dec 2025).

[sklearn] scikit-learn. https://scikit-learn.org/stable/. (accessed 12 Dec 2025).

[XGBoost] XGBoost Documentation. https://xgboost.readthedocs.io/en/stable/. (accessed 12 Dec 2025). [Nogueira 2014] Nogueira F, Bayesian Optimization. Github. 2014. https://github.com/fmfn/BayesianOptimization. (accessed 12 Dec 2025).

[torchmetrics] torchmetrics. https://lightning.ai/docs/torchmetrics/stable/. (accessed 12 Dec 2025).

[McInnes 2018] McInnes L, Healy J, Melville J. Umap: Uniform manifold approximation and projection for dimension reduction. arXiv preprint. 2018;arXiv:1802.03426. https://doi.org/10.48550/arXiv.1802.03426.

Document S2: Bayesian approach estimating virtual time to recurrence for censored patients

In this study, a Bayesian approach was proposed to estimate virtual time to recurrence (TTR) for censored patients to augment the training dataset. The proposed Bayesian approach estimates parameters that determine the TTR distribution according to the reference TTR distribution of a reference paper (124 patients from VU University Medical Center [Senthi 2012]). The estimated parameter distributions were used to calculate the virtual TTR for censored patients in the internal dataset considering their censoring time.

This study assumed the distribution of natural TTR within 5-years to follow the Weibull distribution [Plana 2022]. The density distribution f and survival curve S of recurrence are represented as follows:

$$f(t|\alpha,\lambda) = \alpha\lambda t^{\alpha-1}\exp(-\lambda t^{\alpha}) \quad \text{(S2-1)}$$

$$S(t|\alpha,\lambda) = \exp(-\lambda t^{\alpha}) \quad \text{(S2-2)}$$

where α and λ are shape and scale parameters of the Weibull distribution, t is the TTR, and Eqs. (S2-1) and (S2-2) have a relationship of $f(t) = \frac{dS(t)}{dt}$. To obtain the approximated survival curve $\hat{S}$ based on the reference TTR T, we performed Bayesian estimation of the estimated Weibull parameter distribution $p(\alpha,\lambda|T)$ using a Gibbs sampling method. The estimated parameter distribution $p(\alpha,\lambda|T)$ based on the reference TTR T is expressed as eq. (3) using Bayes' theorem.

$$p(\alpha,\lambda|T) \propto L(T|\alpha,\lambda)\pi(\alpha)\pi(\lambda) \quad \text{(S2-3)}$$

where L is a likelihood function based on the density function f [Cohen 1955], and π is a prior probability function. The prior probability functions of α and λ are defined using a log-normal distribution (Eq. (S2-4)) and gamma distribution (Eq. (S2-5)), respectively.

$$\pi(\alpha) = \sqrt{\frac{\tau}{2\pi}}\frac{1}{\alpha}\exp\left(-\frac{\tau}{2}(ln\,\alpha - \mu)^2\right) \quad \text{(S2-4)}$$

$$\pi(\lambda) = \frac{\delta^\gamma}{\Gamma(\gamma)}\lambda^{\gamma-1}\exp(-\delta\lambda) \quad \text{(S2-5)}$$

$$\Gamma(\gamma) = \int_0^\infty u^{\gamma-1}e^{-u}\ du$$

The Gibbs sampling method is a Markov chain Monte Carlo (MCMC) algorithm for the practical calculation of Bayesian inference. A recursive Markov chain of the Weibull parameters was constructed, and its density distribution was obtained as a posterior distribution $p(\alpha, \lambda|T)$. Supplemental Fig. S2 shows the detailed algorithm of the Gibbs sampling method.

As prior information, we defined the reference TTR T and prior distributions $\pi(\alpha)$ and $\pi(\lambda)$ for the Weibull parameters. The reference TTR distribution T was obtained from a histogram of TTRs in early-stage NSCLC patients treated with SBRT reported in a reference paper [Senthi 2012]. The prior distributions followed Eqs. (S2-4)) and (S2-5). The parameters of prior distributions were $(\mu, \tau, \gamma, \delta) = (0.001, 0.001, 0.001, 0.001)$.

To obtain the estimated Weibull parameter distribution based on the prior information, Gibbs sampling was performed, and a Markov chain was constructed. The Markov chain is defined as the distribution of a sequence of random variables whose next state depends only on the current state. Because of the ergodicity, the Markov chain can construct a stationary distribution (posterior distribution) from any initial value when the chain length is sufficiently long. Therefore, the prior distributions that provided the initial Weibull parameters were defined using arbitrary parameter sets. When the Gibbs sampling is performed J times (= the length of Markov chain), the Markov chain of the Weibull parameters $\{\alpha^{(J)}, \lambda^{(J)}\}$ is expressed as follows:

$$(\alpha_0, \lambda_0), (\alpha_1, \lambda_1), \dots, (\alpha_j, \lambda_j), \dots, (\alpha_J, \lambda_J) \sim \{\alpha^{(J)}, \lambda^{(J)}\}, \qquad (0 \le j \le J) \quad \text{(S2-6)}$$

where j is the iteration number of Gibbs sampling. The initial parameters (α_0, λ_0) are sampled from the prior distributions as $\alpha_0 \sim \pi(\alpha)$ and $\lambda_0 \sim \pi(\lambda)$. When $j \geq 1$, (α_j, λ_j) is sampled in the Gibbs sampling method according to posterior distributions $p(\alpha|\lambda_{j-1}, T)$ and $p(\lambda|\alpha_j, T)$ as follows [Casella 1992]:

$$\alpha_j \sim p(\alpha|\lambda_{j-1}, T) \tag{S2-7}$$

$$\propto \pi(\alpha)\, L(\lambda_{j-1}, t_i \in T|\alpha)$$

$$= \pi(\alpha)\, \prod_{i=1}^{n} \alpha\lambda_{j-1}{t_i}^{\alpha-1}\exp(-\lambda_{j-1}{t_i}^{\alpha})$$

$$\lambda_j \sim p(\lambda|\alpha_j, T) \tag{S2-8}$$

$$\propto \pi(\lambda)\, L(\alpha_j, t_i \in T|\lambda)$$

$$= \pi(\lambda)\, \prod_{i=1}^{n} \alpha_j\lambda{t_i}^{\alpha_j-1}\exp(-\lambda{t_i}^{\alpha_j})$$

α and λ are sampled alternately depending on previous conditions of the parameters as shown in Eqs. (S2-7) and (S2-8), and i represents the censored patient number. The total number of censored patients is n. In this study, the length of Markov chain was determined to $J = 10000$.

Ergodicity is guaranteed when the length of the Markov chain is sufficiently long. However, in practice, the length J is limited for computation of Gibbs sampling. Therefore, a burn-in period was provided to eliminate the influence of fluctuations due to the initial values and early steps of sampling [Hamra 2013]. By constructing the Markov chain excluding the parameters up to the burn-in period b, the variability of posterior distribution can be reduced. In this study, the Markov chain $\{\alpha^{(J')}, \lambda^{(J')}\}$ adjusted with a burn-in period of $b = 1000$, and the joint posterior distribution $p(\alpha, \lambda|T)$ of the parameters α and λ given a reference TTR T was represented as follows:

$$p(\alpha, \lambda|T) \simeq \{\alpha^{(J')}, \lambda^{(J')}\} \qquad (0 \leq j' \leq J'\,;\ j' = j - b) \tag{S2-9}$$

Furthermore, to improve reproducibility, the Markov chain was constructed three times, and the average estimated distributions was considered the estimated Weibull parameter distribution.

We assumed that the virtual TTR $\hat{t}$ could be estimated from the median of the conditional survival curve $S(t|t \geq c, \alpha, \lambda) = 0.5$ [Moghaddam 2022], taking into account the censoring time c of censored patients, as shown in Eq. (S10).

$$\frac{S(\hat{t}_i|\hat{\alpha}_i,\hat{\lambda}_i)}{S(c_i|\hat{\alpha}_i,\hat{\lambda}_i)} = 0.5$$

$$\Longrightarrow \quad \hat{t}_i = \sqrt[\hat{\alpha}_i]{\frac{\ln 2}{\hat{\lambda}_i} + {c_i}^{\hat{\alpha}_i}} \tag{S2-10}$$

$$(\hat{\alpha}_i, \hat{\lambda}_i) \sim \ p(\alpha, \lambda|T) \tag{S2-11}$$

The parameters $(\hat{\alpha}_i, \hat{\lambda}_i)$ for individual censored patients were sampled according to $p(\alpha, \lambda|T)$ (Eq. (S11)) given their censoring times . Censored patients with the virtual TTRs were classified into virtual recurrent (virtual TTR < 5 years; n = 49) and recurrence-free (virtual TTR ≥ 5 years; n = 7) patients. Supplementary Fig. S6 shows a scatter plot of the censoring time and virtual TTR estimated by the Bayesian approach for the censored patients.

References:

[Senthi 2012] Senthi S, Lagerwaard FJ, Haasbeek CJ, Slotman BJ, Senan S. Patterns of disease recurrence after stereotactic ablative radiotherapy for early stage non-small-cell lung cancer: a retrospective analysis. Lancet Oncol. 2012;13(8):802-9. https://doi.org/10.1016/S1470-2045(12)70242-5.

[Plana 2022] Plana D, Fell G, Alexander BM, Palmer AC, Sorger PK. Cancer patient survival can be parametrized to improve trial precision and reveal time-dependent therapeutic effects. Nat Commun. 2022;13(1):873. https://doi.org/10.1038/s41467-022-28410-9.

[Cohen 1955] Cohen Jr AC. Maximum likelihood estimation of the dispersion parameter of a chi-distributed radial error from truncated and censored samples with applications to target analysis. Journal of the American Statistical Association. 1955;50(272):1122-35. https://doi.org/10.1080/01621459.1955.10501295.

[Casella 1992] Casella G, George EI. Explaining the Gibbs Sampler. The American Statistician. 1992;46(3):167–174. https://doi.org/10.1080/00031305.1992.10475878.

[Hamra 2013] Hamra G, MacLehose R, Richardson D. Markov chain Monte Carlo: an introduction for epidemiologists. Int J Epidemiol. 2013;42(2):627-34. https://doi.org/10.1093/ije/dyt043.

[Moghaddam 2022] Moghaddam S, Newell J, Hinde J. A Bayesian Approach for Imputation of Censored Survival Data. Stats. 2022; 5(1):89-107. https://doi.org/10.3390/stats5010006.

Document S3: Selection of significant features

A workflow of significant feature selection is shown in Fig. S5. Significant features that may be associated with time to recurrence (TTR) were selected from important features using hierarchical clustering and importance distance on volcano plots computed from Cox hazard ratio (HR) and Spearman correlation coefficients (SC) [Jin 2025].

Important features were determined on the volcano plot of radiomic features by thresholding the adjusted p-values and either HR or SC. The vertical axis of volcano plot represents $-\log_{10}$(adjusted p-value), and the horizontal axis represents log(HR) or SC. A group of important features IF are defined for the HR and SC methods as follows:

$$IF^{\mathrm{HR}} = \left\{f \in F \mid \left(\mathrm{HR}_f < 0.75 \text{ or } 1.25 < \mathrm{HR}_f\right) \text{ and } p_f < 0.05\right\} \quad \text{(S3-1)}$$

$$IF^{\mathrm{SC}} = \left\{f \in F \mid \left|\mathrm{SC}_f\right| > 0.2 \text{ and } p_f < 0.05\right\} \quad \text{(S3-2)}$$

where p is adjusted p-value of the HR or SC calculated using a Benjamini-Hochberg procedure, and F is a group of extracted radiomic features.

The importance distance of the important feature was computed by the Euclidean distance from the coordinates of the thresholds of the p-value and either HR or SC on the volcano plot the following equations:

$$ID^{\mathrm{HR}}_{f\in IF^{\mathrm{HR}}} = \begin{cases} \sqrt{\left(\log\frac{\mathrm{HR}_f}{1.25}\right)^2 + \left(\log_{10}\frac{0.05}{p_f}\right)^2} & \left(\mathrm{HR}_f > 1.25\right) \\ \sqrt{\left(\log\frac{0.75}{\mathrm{HR}_f}\right)^2 + \left(\log_{10}\frac{0.05}{p_f}\right)^2} & \left(\mathrm{HR}_f < 0.75\right) \end{cases} \quad \text{(S3-3)}$$

$$ID^{\mathrm{SC}}_{f\in IF^{\mathrm{SC}}} = \begin{cases} \sqrt{\left(\mathrm{SC}_f - 0.2\right)^2 + \left(\log_{10}\frac{0.05}{p_f}\right)^2} & \left(\mathrm{SC}_f > 0.2\right) \\ \sqrt{\left(\mathrm{SC}_f + 0.2\right)^2 + \left(\log_{10}\frac{0.05}{p_f}\right)^2} & \left(\mathrm{SC}_f < -0.2\right) \end{cases} \quad \text{(S3-4)}$$

The significant features SF were most important feature in each hierarchical cluster determined by the longest importance distance. Hierarchical clustering was performed using the Ward D2 algorithm based on the squared Euclidean distance to remove redundancy among the significant features [proxy]. The number of clusters m ($m = 6$ in this study) was the same as that of the significant features. Eq. 5 represents the significant feature selection from each cluster using importance distance.

$$SF_k = \left\{ \underset{f \in IF \cap Cluster_k}{\operatorname{argmax}} ID_f \mid k = 1, \dots, m \right\} \tag{S3-5}$$

References:

[Jin 2025] Jin Y, Arimura H, Iwasaki T, Kodama T, Yamamoto N, Cui Y, et al. Multiscale Fusion Models With Genomic, Topological, and Pathomic Features to Predict Response to Radiation Therapy for Non-Small Cell Lung Cancer Patients. Lab Invest. 2025;105(10):104204. https://doi.org/10.1016/j.labinv.2025.104204.

[proxy] proxy. https://cran.r-project.org/web/packages/proxy/proxy.pdf. (accessed 12 Dec 2025).

Document S4: Conventional early recurrence prediction models

Cox regression [CoxPHFitter] and random survival forest (RSF) models [RandomSurvivalForest] without proposed Bayesian approach were also implemented as conventional methods for the early (1- or 2-year) recurrence prediction. The early recurrence was directly predicted using the six significant original features selected in the Subsection 2.9. The hyperparameters (Table S6) were optimized by maximizing the robustness index (RI_{auc}) of the areas under the receiver operating characteristic (ROC) curves (AUCs) in 100 iterations of Bayesian optimization [Nogueira 2014]. The RI_{auc} was calculated from the AUCs in the training AUC_{train} and validation AUC_{valid} as follows:

$$RI_{auc} = \frac{AUC_{valid}}{1+|AUC_{train}-AUC_{valid}|} \qquad \text{(S4-1)}$$

The most robust combination of the conventional model and the selection model was determined by the mean AUC across all three folds of the internal test dataset (Tables 2, S8 and S9).

References:

[CoxPHFitter] CoxPHFitter. https://lifelines.readthedocs.io/en/latest/fitters/regression/CoxPHFitter.html. (accessed 04 Jun 2026)

[RandomSurvivalForest] RandomSurvivalForest. https://scikit-survival.readthedocs.io/en/stable/api/generated/sksurv.ensemble.RandomSurvivalForest.html. (accessed 04 Jun 2026).

[Nogueira 2014] F. Nogueira, Bayesian Optimization. Github. 2014. https://github.com/fmfn/BayesianOptimization. (accessed 12 Dec 2025).
